# *PcMuORP1*, an Oxathiapiprolin-Resistance Gene, Functions as a Novel Selection Marker for *Phytophthora* Transformation and CRISPR/Cas9 Mediated Genome Editing

**DOI:** 10.3389/fmicb.2019.02402

**Published:** 2019-10-22

**Authors:** Weizhen Wang, Zhaolin Xue, Jianqiang Miao, Meng Cai, Can Zhang, Tengjiao Li, Borui Zhang, Brett M. Tyler, Xili Liu

**Affiliations:** ^1^Department of Plant Pathology, College of Plant Protection, China Agricultural University, Beijing, China; ^2^State Key Laboratory of Crop Stress Biology for Arid Areas, College of Plant Protection, Northwest A&F University, Yangling, China; ^3^Key Laboratory of Pesticide and Chemical Biology of Ministry of Education, College of Chemistry, Central China Normal University, Wuhan, China; ^4^Center for Genome Research and Biocomputing, Oregon State University, Corvallis, OR, United States; ^5^Department of Botany and Plant Pathology, Oregon State University, Corvallis, OR, United States

**Keywords:** oomycetes, *Phytophthora*, transformation, selection marker, oxathiapiprolin-resistance, CRISPR/Cas9

## Abstract

*Phytophthora*, a genus of oomycetes, contains many devastating plant pathogens, which cause substantial economic losses worldwide. Recently, CRISPR/Cas9-based genome editing tool was introduced into *Phytophthora* to delineate the functionality of individual genes. The available selection markers for *Phytophthora* transformation, however, are limited, which can restrain transgenic manipulation in some cases. We hypothesized that *PcMuORP1*, an endogenous fungicide resistance gene from *P. capsici* that confers resistance to the fungicide oxathiapiprolin via an altered target site in the ORP1 protein, could be used as an alternative marker. To test this hypothesis, the gene *PcMuORP1* was introduced into the CRISPR/Cas9 system and complementation of a deleted gene in *P. capsici* was achieved using it as a selection marker. All of the oxathiapiprolin-resistant transformants were confirmed to contain the marker gene, indicating that the positive screening rate was 100%. The novel selection marker could also be used in other representative *Phytophthora* species including *P. sojae* and *P. litchii*, also with 100% positive screening rate. Furthermore, comparative studies indicated that use of *PcMuORP1* resulted in a much higher efficiency of screening compared to the conventional selection marker *NPT II*, especially in *P. capsici.* Successive subculture and asexual reproduction in the absence of selective pressure were found to result in the loss of the selection marker from the transformants, which indicates that the *PcMuORP1* gene would have little long term influence on the fitness of transformants and could be reused as the selection marker in subsequent projects. Thus, we have created an alternative selection marker for *Phytophthora* transformation by using a fungicide resistance gene, which would accelerate functional studies of genes in these species.

## Introduction

The oomycetes are a class of eukaryotic microorganisms that have similar life cycles and growth habits to the filamentous fungi. Oomycota contains many pathogens of both plants and animals (Kamoun et al., 2015; Derevnina et al., 2016) including the infamous *Phytophthora infestans* that was responsible for the Irish potato famine of the nineteenth century and which remains a severe threat to potato production to this day (Zadoks, 2008; Fry et al., 2015). Although the oomycetes share many biological characteristics with the true fungi, they are phylogenetically distinct, belonging to the kingdom Stramenopila, which also includes the golden-brown algae, diatoms, and brown algae (Baldauf et al., 2000; Yoon et al., 2002; Tyler et al., 2006). In most instances, the oomycetes differ from fungi in many ways including their genome size, ploidy of vegetative hyphae, cell wall composition (cellulose instead of chitin), and the type of mating hormones produced (Judelson and Blanco, 2005). Due to lower transformation efficiency and homologous recombination rate, it is more difficult to genetically manipulate oomycetes than the most of amenable true fungi (Judelson, 1997).

The oomycete genus, *Phytophthora*, is of great importance economically and ecologically. For example, it was estimated that worldwide vegetable production valued at over one billion dollars is threatened by *P. capsici* alone each year (Lamour et al., 2012). To investigate the unique biology of *Phytophthora*, different classical and molecular genetic strategies have been adopted, with gene silencing via RNA interference (RNAi) being most commonly used in functional studies (van West et al., 1999; Dou et al., 2010; Liu et al., 2011; Wang et al., 2011; Lu et al., 2013; Tyler and Gijzen, 2014). Despite these successes, the RNAi strategy has several drawbacks when applied to *Phytophthora* species and some other organisms. For examples, the degree of gene silencing is unpredictable, and some of the transformants can be unstable. Furthermore, it is difficult to reliably silence several unrelated genes simultaneously (Bargmann, 2001; Kamath et al., 2001; Hannon, 2002; Alonso et al., 2003). The recent development of the clustered regularly interspaced short palindromic repeat (CRISPR)/CRISPR-associated protein 9 nuclease (Cas9) system which has been employed for a variety of genome editing procedures in a wide range of organisms (Xie et al., 2015), is therefore extremely exciting. In 2016, the CRISPR/Cas9 system was successfully applied in *P. sojae* (Fang and Tyler, 2016) for the first time, and subsequently in *P. palmivora* (Gumtow et al., 2018), indicating that this method might have broad application in the genus *Phytophthora*.

Genetic modification of *Phytophthora* spp. requires specialized vectors and can be achieved by several transformation protocols including PEG-CaCl_2_-mediated protoplast transformation, *Agrobacterium*-mediated transformation, electroporation and microprojectile bombardment (Judelson and Michelmore, 1991; Cvitanich and Judelson, 2003; Latijnhouwers and Govers, 2003; Vijn and Govers, 2003; Wu et al., 2016). Selection markers play a critical role in any transformation methodology as they allow the transformed cells to be distinguished from non-transformed ones, with antibiotic resistance genes and auxotrophic markers being widely used for screening transformants of microorganisms (Kanda et al., 2014). However, the range of selection markers available for *Phytophthora* transformation is severely limited for the moment. Indeed, many species depend on just one, the *NPT II* gene, which encodes the neomycin phosphotransferase II enzyme that confers resistance to kanamycin, neomycin and geneticin (G418) (Bashir et al., 2016). The hygromycin B resistance gene *HPT*, which encodes the hygromycin phosphotransferase, was also frequently used in *P. infestans*, whereas it was inefficient in some other *Phytophthora* species such as *P. capsici* and *P. parasitica*, as very high concentrations of hygromycin B (at least 400 μg/ml) were required to completely inhibit the background growth (Bailey et al., 1991; van West et al., 1999). Furthermore, the use of *HTP* in *P*. *sojae* resulted in very few transformants being recovered (Judelson et al., 1993), which indicates that is not efficient for the transformation of *P*. *sojae* either. The lack of appropriate selection markers has hampered the progress of functional gene studies in *Phytophthora*, and the development of alternative selection makers is crucial for complementation experiments and multiplex editing of genome.

The anti-oomycete compound oxathiapiprolin, which was developed by DuPont in 2007, exhibits extremely high activity against most plant pathogenic oomycetes, including *P. capsici*, *P. sojae*, *P. litchii*, and *P. nicotianae*, as well as *Pseudoperonospora cubensis* amongst others (Ji et al., 2014; Miao et al., 2016b). The mode of action of oxathiapiprolin has been well characterized in oomycetes, with the primary target protein being the oxysterol binding protein (OSBP), which is a member of the OSBP-related proteins (ORPs) family (Pasteris et al., 2016). However, resistance to oxathiapiprolin has recently been documented in *Phytophthora*, with point mutations in the ORP1 protein resulting in high level of resistance (Miao et al., 2016a, 2018). Although this discovery was worrying from a crop protection perspective, it provided a possible opportunity to develop an alternative selection marker for *Phytophthora* transformation. The current study was initiated to determine whether a mutated version of the *PcORP1* sequence (*PcMuORP1*) could be used as a reliable selection marker in *P. capsici*, and if so, whether its use could be extended to other *Phytophthora* species. In addition, the screening efficiency of this novel marker was compared with that of the traditional marker *NPT II*.

## Materials and Methods

### *Phytophthora* Isolates and Growth Conditions

The *P. capsici* isolate BYA5 was collected from an infected pepper sample in Gansu Province of China in 2011, while the *P. litchii* isolate ZB2-4 was collected from infected lychee in Guangdong Province in 2014. The *P. sojae* isolate P6497 has been widely used in previous oomycete studies and its entire genome has been sequenced and published (Tyler et al., 2006).

The three different *Phytophthora* isolates were all maintained on solid V8 medium at 25°C in the dark. Zoospores of *P. sojae* were prepared according to a method described by Ward et al. (1989), with a few modifications. The mycelia from 15 ml cultures grown in clarified V8 media inoculated with 10 mycelial plugs were harvested after 3 days’ dark-incubation at 25°C, and washed 5 times with sterile water at 20 min intervals. The collected mycelium was then submerged in 10 ml of sterile water and dark-incubated at 25°C for 8–10 h to facilitate sporangium production and zoospore release. A different method based on the protocol from a previous study (Pang et al., 2013) was used to produce zoospores of *P. capsici*. In this case *P. capsici* isolates were dark-incubated at 25°C on solid V8 medium for 3–4 days before being switched to a 12 h-light/12 h-dark photoperiod for an additional 6 days. The plates were then flooded with 10 ml sterile water and incubated at 4°C for 30 min, and then at room temperature for 30 min.

The zoospores of both *P. sojae* and *P. capsici* were plated on the surface of 1% agar plates and dark-incubated at 25°C for 3–8 h until most of them had germinated. Single spore purification was achieved by transferring individual germinated spores to fresh V8 plates, which were then dark-incubated at 25°C for 3 days.

### Fungicide and Antibiotics

Technical grade oxathiapiprolin (96.7%, active ingredient) was kindly provided by DuPont Crop Protection (Wilmington, DE, United States), and the ultra-pure grade G418 sulfate and ampicillin sodium salt were purchased from Amresco (Solon, OH, United States), while hygromycin B solution (50 mg/ml) was purchased from QiDi Taili Technology (Beijing, China).

A range of oxathiapiprolin stock solutions were prepared in dimethyl sulfoxide (0.25, 0.2, 0.1, 0.05, 0.01, 0.005, 0.0025, 0.00125, and 0.000625 mg/ml) and stored in the dark at 4°C until required. The antibiotics G418, hygromycin B, and ampicillin were dissolved or diluted in ultra-pure grade water to prepare stock solutions (50, 30, 15, 7.5, 3.75, and 1.875 mg/ml for G418; 50, 25, 12.5, 6.25, and 3.125 mg/ml for hygromycin B; 100 and 50 mg/ml for ampicillin), which were stored in the dark at −20°C.

### Nucleic Acid Isolation From *Phytophthora* Species

The mycelia from 4 days old plate cultures of *P. capsici* isolate BYA5 were harvested and frozen at −80°C until required. Total RNA was extracted from the frozen samples using the SV Total RNA Isolation kit (Promega, Beijing, China) and cDNA was synthesized using the PrimeScript RT reagent Kit with gDNA Eraser (Takara, Beijing, China) according to the protocols of the manufacturers. The DNA isolation from the *P. capsici*, *P. sojae*, and *P. litchii* wild-type isolates as well as transformants was performed using mycelia that had been harvested from 4 to 7 days old cultures and frozen at −20°C. The total DNA was then isolated using the method detailed in a previous study (Ristaino et al., 1998). All the cDNA and DNA samples were stored at −20°C until required.

### MICs Determination

The *P. capsici* isolate BYA5, *P. sojae* isolate P6497 and *P. litchii* isolate ZB2-4 were dark-incubated at 25°C for 3 days, and mycelial plugs taken from the periphery of actively growing colonies were transferred to fresh V8 agar modified with series of concentration of fungicide and antibiotics (0.25, 0.2, 0.1, 0.05, 0.01, 0.005, 0.0025, 0.00125, and 0.000625 μg/ml for oxathiapiprolin, 50, 30, 15, 7.5, 3.75, and 1.875 μg/ml for G418; 100, 50, 25, 12.5, 6.25, and 3.125 μg/ml for hygromycin B), and 3 replicates were made for each treatment. After 3 days’ dark-incubation at 25°C, the mycelial growth was checked. The lowest concentrations that totally inhibited the mycelial growth were considered as minimum inhibitory concentrations (MICs).

### Transformation of *Phytophthora* Species

The plasmids described in the current study were used to transform the different *Phytophthora* species using the PEG-CaCl_2_ mediated protoplast method described previously (Dou et al., 2008; Fang and Tyler, 2016), but with a few modifications. Briefly, 2 days old *P. capsici* (or 3 days old *P. sojae*, or 2 days old *P. litchii*) mycelial mats cultured in liquid pea broth medium, were rinsed with ultra-pure water and suspended in 0.8 M mannitol with gentle shaking. After 10 min incubation at 25°C the mycelia were transferred to 20 ml of enzyme solution [0.4 M mannitol, 20 mM KCl, 20 mM MES, pH 5.7, 10 mM CaCl_2_, 0.75% Lysing Enzymes from *Trichoderma harzianum* (Sigma L1412: St Louis, MO, United States) and 0.75% Cellulysin^®^ Cellulase (Calbiochem 219466: San Diego, CA, United States)] and incubated at room temperature for approximately 35 min (40 min for *P. sojae*, 35 min for *P. litchii*) with gentle shaking (55–60 rpm). The mixture was then filtered through two layers of Miracloth (EMD Millipore Corp., 2913897: Billerica, MA, United States) and the resulting protoplasts were collected by centrifugation at 530 g for 4 min in a 50 ml centrifuge tube. After being washed with 30 ml of W5 solution (5 mM KCl, 125 mM CaCl_2_, 154 mM NaCl and 173 mM glucose), the protoplasts were resuspended in 5–10 ml of W5 solution and placed on ice for 30 min before being collected by centrifugation at 530 g for 5 min in a 50 ml centrifuge tube and resuspended at 10^6^–10^7^/ml in MMg solution (0.4 M mannitol, 15 mM MgCl_2_ and 4 mM MES, pH 5.7). The transformation itself was performed using 1 ml of protoplast suspension with 40–50 μg of DNA for single plasmid transformations or 20–30 μg of DNA for each plasmid for co-transformations. The protoplast-plasmid mixtures were incubated on ice for 20–30 min, before 1740 μl of fresh PEG solution (40% PEG 4000 m/v, 0.2 M mannitol and 0.1 M CaCl_2_) was gradually added. The tubes were then shaken gently and incubated on ice for another 20–30 min before the protoplasts were mixed with 20 ml of pea broth containing 0.5 M mannitol and dark-incubated at 18°C for 20–24 h to allow regeneration.

### Screening of *Phytophthora* Transformants

The regenerated protoplasts were collected by centrifugation at 700 g for 5 min, and resuspended in 35 ml of molten (42–45°C) pea broth agar (1.5% agar, 0.5 M mannitol) amended with the appropriate selective agent: 0.005 μg/ml (or higher) oxathiapiprolin or 30 μg/ml G418 when *NPT II* was used as the selection marker. The medium containing the regenerated protoplasts was then divided among three Petri dishes and dark-incubated at 25°C for 3 days to complete the first round of screening. Each plate was then covered with molten (42–45°C) V8 medium (1.5% agar) amended with a higher dose of the selective agents: 0.01 μg/ml (or higher) for oxathiapiprolin or 50 μg/ml for G418, and incubated for a further 3 days under the same conditions. The colonies that appeared after this second round of screening were transferred to fresh V8 plates amended with 0.01 μg/ml (or higher) oxathiapiprolin or 50 μg/ml G418 for the third round of screening. 50–100 μg/ml ampicillin could be used to avoid bacterial contamination in each screening step.

### PCR Analysis and Sequence Alignment

All the polymerase chain reactions conducted in the current study, except those performed during plasmid construction, were performed using *EasyTaq* DNA Polymerase and 25 μl reaction mixtures containing 12.5 μl 2x Master Mix (Tsingke, Beijing, China), 0.5 μl Forward Primer (10 μM), 0.5 μl Reverse Primer (10 μM), and 500–1000 ng template DNA. The PCR itself was conducted using a Bio-gener GT9612 thermocycler (Bio-gener, Hangzhou, China) and the following program: initial denaturing at 94°C for 4 min, followed by 34 cycles of denaturing at 94°C for 30 s, annealing at 55–62°C (depending on the primer) for 30 s and extension at 72°C for 1 min for each 1 kb fragment, with a final extension at 72°C for 10 min. The resulting PCR products were visualized by agarose gel electrophoresis and sequenced by Tsingke (Beijing, China). Multiple sequence alignments were prepared using the DNAMAN 9.0.1.116 software package (Lynnon Biosoft, Quebec, QC, Canada).

### Statistical Analysis

The data collected in the current study were subjected to analysis of variance using DPS software ver. 7.05 (Zhejiang University, Hangzhou, China). Differences between means were determined using Duncan’s multiple range test at *P* = 0.05.

## Results

### Gene Complementation in *P. capsici* Using *PcMuORP1* as the Selection Marker

The oxathiapiprolin target protein PcORP1 consists of 957 amino residues. Previous research has shown that a single point mutation at residue 769 (G769W), which corresponds a nucleotide substitution of guanine to thymine at nucleotide 2305 (G2305T) in the coding DNA sequence (CDS), can result in high level of fungicide resistance in *P. capsici* (Miao et al., 2016a). The G2305T mutation was successfully introduced into the *PcORP1* CDS derived from cDNA (Supplementary Methods S1), and the mutated version of *PcORP1* CDS, was hereafter referred to as *PcMuORP1*.

The potential of *PcMuORP1* as a selection marker was initially evaluated via gene complementation. For this purpose, the *PcDHCR7* gene of *P. capsici*, which encodes a homolog of 7-dehydrocholesterol reductase in the sterol synthesis pathway (Desmond and Gribaldo, 2009), was first replaced by the G418 resistance gene *NPT II* (Supplementary Methods S2). The resultant isolate KD1-1 was a homozygous transformant in which the *NPT II* gene replaced the *PcDHCR7* gene and was thus insensitive to G418. Therefore, further genetic manipulation of KD1-1 was impossible without the use of an alternative selection marker. Thus we used KD1-1 as a rigorous model to test the potential of *PcMuORP1* as a selection marker for CRISPR/Cas9-mediated gene complementation in *P. capsici*.

**FIGURE 1:**
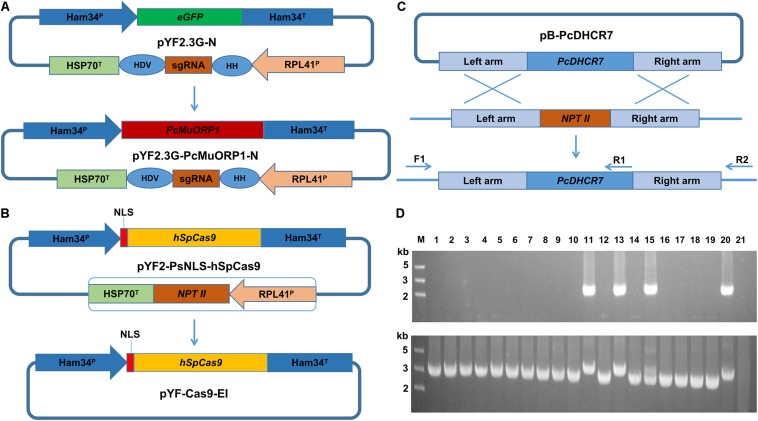
Plasmid modification and complementation of the *PcDHCR7* gene. **(A)** Replacement of the *eGFP* gene in pYF2.3G-N by the fungicide resistance gene *PcMuORP1* to generate the novel plasmid pYF2.3G-PcMuORP1-N. HH, hammerhead (HH) ribozyme; HDV, HDV ribozyme. **(B)** Removal of the *NPT II* expression cassette from pYF2-PsNLS-hSpCas9 to form pYF-Cas9-EI. **(C)** Schematic representation of the homologous recombination that restored the *PcDHCR7* gene in place of the *NPT II* gene in *P. capsici* isolate KD1-1, showing the location of the primers used to validate complementation had occurred and to differentiate heterozygous and homozygous transformants. F1, ReD-F Forward primer; R1, ReD-R1 Reverse primer; R2, ReD-R2 Reverse primer. **(D)** DNA electrophoresis of PCR products amplified from transformants. Top: The F1/R1 primer set confirms complementation has occurred in lanes 11, 13, and 15. Bottom: The F1/R2 primer set confirms homozygous complementation in lanes 11 and 13. M, DNA Marker; Lanes 1–18, oxathiapiprolin-resistant transformants; Lane 19, Parental isolate KD1-1 used as a negative control; Lane 20, Wild-type isolate BYA5 used as a positive control; Lane 21, Blank control.

For the gene complementation, three novel plasmids, pYF2.3G-PcMuORP1-N (Modified from plasmid pYF2.3G-N, Figure 1A and Supplementary Methods S1), which contained the sgRNA and the selection marker *PcMuORP1*; pYF-Cas9-EI (Simplified from vector pYF2-PsNLS-hSpCas9 by removal of the *NPT II* gene, Figure 1B and Supplementary Methods S1), which expressed the Cas9 protein; and pB-PcDHCR7 (Created from vector pBluescript II KS+, Figure 1C and Supplementary Methods S1), which served as the homologous replacement template, were simultaneously transformed into protoplasts of KD1-1. After the transformation, putative complementation transformants were selected using oxathiapiprolin, which yielded hundreds of resistant colonies. PCR using *PcDHCR7-*specific primers (ReD-F and ReD-R1, Supplementary Table S2) was then conducted to further screen the oxathiapiprolin-resistant transformants to identify those that had been successfully complemented. A further round of PCR was conducted to distinguish homozygous transformants on the basis that the *PcDHCR7* gene (1367 bp) is longer than the *NPT II* gene (795 bp). A total of 18 independent oxathiapiprolin-resistant transformants, each of which had undergone single spore purification, were evaluated using the parental isolate KD1-1 as the negative control and the wild-type isolate BYA5 as the positive control.

DNA electrophoresis revealed that three of the 18 oxathiapiprolin-resistant transformants had been successfully complemented (lanes 11, 13, and 15, Figure 1D), two of which were homozygous (lanes 11 and 13, Figure 1D). Gene sequencing of the complemented transformants showed that the genome editing was totally accurate. In addition, the *PcMuORP1* gene were also successfully used as the selection marker in knock-out projects of different genes such as *PcERG3*, which encodes a homolog of sterol C5 desaturase in *P. capsici* (Desmond and Gribaldo, 2009; Supplementary Methods S2 and Supplementary Figure S1). Taken together these results validated the hypothesis that the oxathiapiprolin-resistance gene *PcMuORP1* could be used as an alternative selection marker during *P. capsici* transformation, and also demonstrated that site-specific complementation of gene deletions could be achieved using the modified CRISPR/Cas9 system. For convenience of sgRNA expressing plasmid construction, an all-in-one backbone plasmid pYF515-PcMuORP1 (Supplementary Table S1) containing PcMuORP1, Cas9 and sgRNA expressing cassettes was created (Supplementary Methods S1 and Supplementary Figure S2).

### All Oxathiapiprolin-Resistant Colonies Contained the *PcMuORP1* Transgene

**FIGURE 2:**
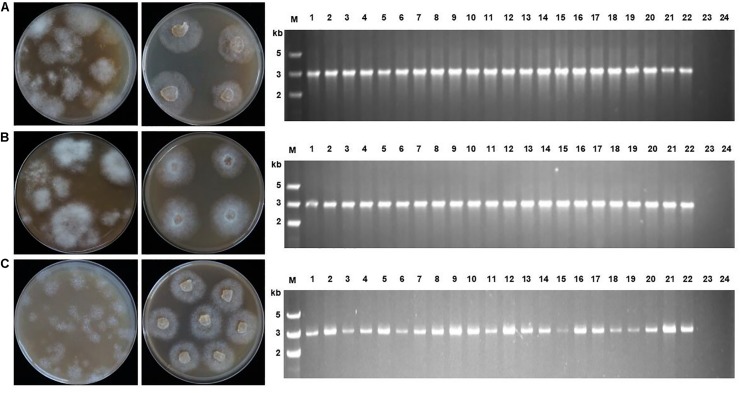
Transformant screening on oxathiapiprolin-amended media, and PCR confirmation that transformants contained the *PcMuORP1* selection marker. **(A)** Three plasmids co-transformation for *P. capsici.* **(B)** One plasmid transformation for *P. sojae.* **(C)** One plasmid transformation for *P. litchii.* Left: Colonies resulting from the second screening with V8 medium containing 0.2 μg/ml oxathiapiprolin in the case of *P. capsici*, and 0.01 μg/ml in *P. sojae* and *P. litchii*. Middle: Colonies resulting from the third screening on V8 medium containing 0.25 μg/ml oxathiapiprolin in the case of *P. capsici*, and 0.01 μg/ml in *P. sojae* and *P. litchii*. Right: PCR confirming 22 randomly selected transformants contained the *PcMuORP1* selection marker. Lanes 1–22, oxathiapiprolin-resistant transformants; Lane 23, Parental isolates used as negative controls; Lane 24, Blank control.

A three-step screening process was used to select oxathiapiprolin-resistant transformants using increasing concentrations of oxathiapiprolin (0.005–0.25 μg/ml). When the regenerated protoplasts were initially plated in pea broth agar, 0.005 μg/ml oxathiapiprolin was used. After 2 or 3 days, mycelia were observed emerging from the pea broth agar. At that point, the medium was overlaid with molten V8 agar, amended with 0.2 μg/ml oxathiapiprolin. After 2–3 days further incubation, a multitude of discrete colonies appeared. Those colonies were transferred to fresh V8 agar (with 0.25 μg/ml oxathiapiprolin), and all of them were observed to continue to grow well with hardly any inhibition (Figure 2A). This suggests that, in future, an even higher concentration of oxathiapiprolin could be used to increase the stringency of the screening process, if necessary.

PCR was conducted to validate whether the transformants selected during the screening process contained functional copies of the *PcMuORP1* selection marker. The primers used were designed to avoid false-positives amplified from the endogenous *PcORP1* gene, with the forward primer Resg-ORP1-F being located in the Ham34 promoter, and the reverse primer Resg-ORP1-R within the *PcMuORP1* gene itself (Supplementary Table S2). We tested 22 randomly selected transformants from the three-step screening process used in the complementation experiment described above. The results of the PCR analysis were definitive, with all of the 22 transformants yielding PCR products of the correct length, whereas the parental isolate KD1-1 used as a negative control, did not yield any product. Thus, all of the 22 *P. capsici* transformants, but not the parental isolate KD1-1, harbored the *PcMuORP1* resistance gene (Figure 2A). Taken together these results provide clear confirmation that the combination of oxathiapiprolin and the *PcMuORP1* resistance gene provides an extremely effective method for selecting transformants in *P. capsici*, producing a successful screening rate of 100%.

### The Application of *PcMuORP1* as a Selection Marker in Other *Phytophthora* Species

The potential of the oxathiapiprolin-resistance gene *PcMuORP1* as a selection marker for the transformation of other *Phytophthora* spp. was investigated using two representative species, *P. sojae* and *P. litchii*. The first, *P. sojae*, is a destructive pathogen of soybean that can cause substantial economic losses to oil crop production and has emerged as a model oomycete species for several areas of research (Tyler, 2007). The second species, *P. litchii*, causes downy blight disease in lychee resulting in significant damage to fruit production (Kao and Leu, 1980; Jiang et al., 2017). The investigation was initially focused on *P. sojae* isolate P6497, which was transformed using the pYF2.3G-PcMuORP1-N plasmid carrying the oxathiapiprolin-resistance gene *PcMuORP1*. The results of the transformation were similar to those obtained in *P. capsici*, with hundreds of oxathiapiprolin-resistant transformants being recovered from the three-step screening process (Figure 2B). A range of different oxathiapiprolin concentrations (0.005–0.1 μg/ml) were evaluated, with even the lowest concentration providing effective screening. Furthermore, PCR analysis of 22 randomly selected oxathiapiprolin-resistant *P. sojae* transformants confirmed that all of them contained the *PcMuORP1* transgene (Figure 2B), indicating that *PcMuORP1* is an effective selection marker for the transformation of *P. sojae* with a successful screening rate of 100%. Identical results were obtained when exactly the same experiments were performed with *P. litchii* (Figure 2C). Taken together, these results indicate that *PcMuORP1* could be an extremely effective selection marker for the transformation of a broad range of *Phytophthora* species.

### Relative Screening Efficiency of the Selection Markers *PcMuORP1* and *NPT II*

Having established that *PcMuORP1* could be successfully used as a selection marker for the transformation of a range of *Phytophthora* species, a further series of experiments were conducted to compare the screening efficiency obtained with *PcMuORP1* gene relative to the conventional selection marker *NPT II*. The first step in the investigation was to construct an *NPT II* plasmid equivalent to the pYF2.3G-PcMuORP1-N plasmid (Supplementary Methods S1), where the selection marker *NPT II* would be under the control of the same promoter, Ham 34. The investigation was conducted using the three *Phytophthora* species evaluated previously, with parallel experiments being conducted to transform the two plasmids pYF2.3G-PcMuORP1-N and pYF2.3G-NPT-N into the protoplasts of *P. capsici*, *P. sojae*, and *P. litchii.*

Each transformation experiment involved introducing equivalent quantities of the plasmids to 1 ml samples of protoplasts, which were standardized to the following concentrations: 4.5 × 10^6^ per milliliter for *P. capsici*, and 1.5 × 10^6^ per milliliter for *P. sojae* and *P. litchii*. The resulting transformants were screened with oxathiapiprolin in the case of pYF2.3G-PcMuORP1-N and G418 in the case of pYF2.3G-NPT-N. Given that the selective pressure used in early screening stage may influence the transformants recovered, we determined the minimal inhibitory concentrations (MICs) of oxathiapiprolin and G418, as well as hygromycin B, for the three *Phytophthora* species (Table 1). For the screening efficiency comparison, the concentrations of oxathiapiprolin and G418 for first round of screening were set to 0.005 and 30 μg/ml, respectively. In this way, the early selective pressure of oxathiapiprolin was almost the same for *P. sojae*, and higher for *P. capsici*, and lower for *P. litchii*, compared with that of G418 (Table 1).

**TABLE 1:** MICs determination of oxathiapiprolin and two antibiotics to three *Phytophthora* spp.

***Phytophthora* sp.**	**Isolate**	**MIC (μg/ml)^a^**		
		**Oxathiapiprolin**	**G418**	**Hygromycin B**
*P*. *capsici*	BYA5	0.00125 (4)	15 (2)	>100
*P*. *sojae*	P6497	0.0025 (2)	15 (2)	25
*P*. *litchii*	ZB2-4	0.00125 (4)	3.75 (8)	25

*^*a*^The MICs were determined by mycelium growth evaluation on V8 medium modified by series of concentration of fungicide and antibiotics; selective pressure factors relative to MICs are shown in parentheses when 0.005 μg/ml oxathiapiprolin (or 30 μg/ml G418) was used for transformant selection.*

**FIGURE 3:**
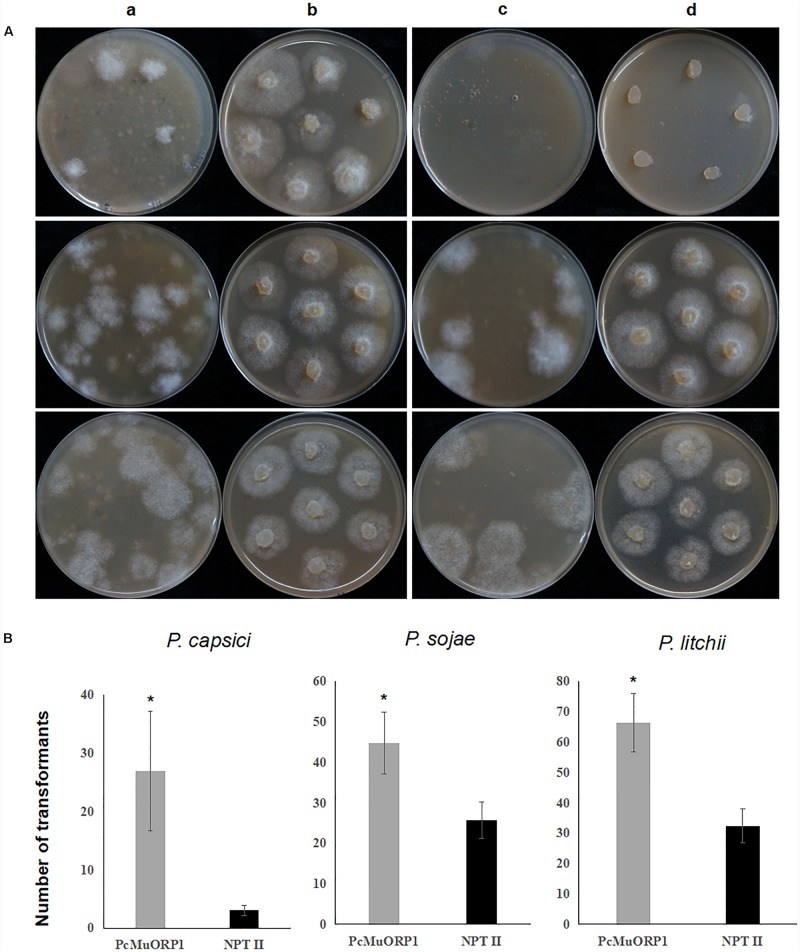
Screening efficiency in three *Phytophthora* species using two different selection markers, *PcMuORP1* and *NPT II*. **(A)** Transformant colonies of *P. capsici* (top), *P. sojae* (middle) and *P. litchii* (bottom) screened using either oxathiapiprolin or G418, respectively. a: Transformant colonies (*PcMuORP1*) after 3 days of culture on V8 medium amended with 0.01 μg/ml oxathiapiprolin during the second screening, b: Subcultured colonies from the second screening after 2 days (3 days for *P. sojae* and *P. litchii*) of culture on V8 medium amended with 0.01 μg/ml oxathiapiprolin during the third screening, c: Transformant colonies (*NPT II*) after 3 days of culture on V8 medium modified with 50 μg/ml G418 during the second screening, d: Subcultured colonies from the second screening after 2 days (3 days for *P. sojae* and *P. litchii*) of culture on V8 medium modified with 50 μg/ml G418 during the third screening. **(B)** Numbers of transformants recovered from 1 ml of protoplasts (4.5 × 10^6^ per milliliter for of *P. capsici*; 1.5 × 10^6^ per milliliter for *P. sojae* and *P. litchii*). Values represent the mean ± SD of three biological replicates derived from three independent experiments (*n* = 4), and asterisks denote significant difference from *NPT II*. ^∗^*p* < 0.05.

The screening efficiency was evaluated by calculating the number of resistant transformants obtained from the two different plasmids. Four technical replicates were prepared for each treatment and the entire experiment was conducted three times using different batches of plasmid DNA. The numbers of transformants resulting from the three-step screening process were counted from each treatment and the data collected were subjected to analysis of variance to determine statistical differences between the treatments. The results clearly indicated that *PcMuORP1* increased screening efficiency relative to *NPT II*, producing a higher number of transformants in all three *Phytophthora* species (Figure 3). This effect was particularly pronounced in *P. capsici*, which yielded very few transformants when *NPT II* was used as the selection marker. Even though the current study validated *NPT II* as an effective selection marker for the transformation of *P. sojae* and *P. litchii*, it also showed that *PcMuORP1* could almost double the efficiency of screening in these species (Figure 3).

### Stability of *PcMuORP1* in Regenerated Transformants

**FIGURE 4:**
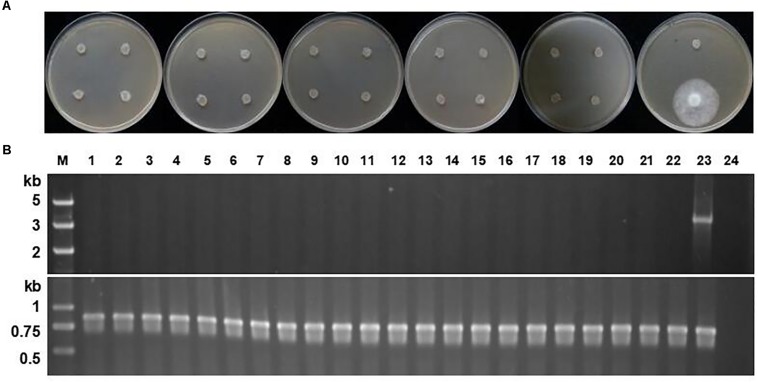
Stability of the *PcMuORP1* selection marker in *P. sojae.* **(A)** Growth of the 21 *P. sojae* transformants from 10 successive rounds of vegetative subculture in the absence of selection pressure on V8 media amended with 0.01 μg/ml oxathiapiprolin after 3 days dark-incubation at 25°C. The last isolate which could grow in the presence of oomyceticide was the one maintained on oxathiapiprolin-amended V8 medium. **(B)** PCR analysis for the detection of the *PcMuORP1* gene. Top: *PcMuORP1*-specific primers. Bottom: Actin-specific primers. Lanes 1–21, 21 *P. sojae* transformants after 10 successive rounds of vegetative subculture in the absence of selection pressure; Lane 22, Wild-type isolate P6497 used as a negative control; Lane 23, *P. sojae* transformant maintained on oxathiapiprolin-amended V8 medium; Lane 24, Blank control.

Once the screening process is completed, the selection marker becomes obsolete and sometimes reduces the fitness of transformants (Scutt et al., 2002). The stability of the selection marker in *P. sojae* was evaluated by subjecting 21 of the 22 oxathiapiprolin-resistant *P. sojae* transformants described above to 10 rounds of vegetative subculture on V8 plates in the absence of oxathiapiprolin, with the other isolate maintained on oxathiapiprolin-amended V8 medium, as a positive control. In addition, single zoospore progeny from 8 of the same 22 transformants (4 spores from each of the 8 transformants), were assessed for oxathiapiprolin resistance, while the parental transformants maintained on oxathiapiprolin-amended V8 medium were used as positive controls. The passaged cultures and the single zoospore isolates were evaluated for growth inhibition on oxathiapiprolin-amended V8 media (0.01 μg/ml oxathiapiprolin), and by PCR analysis to determine whether they still contained the *PcMuORP1* transgene. Two primer pairs were used for the PCR assay: Resg-ORP1-F/Resg-ORP1-R, which were specific to the *PcMuORP1* transgene, and Ps-Actin-F/Ps-Actin-R (Supplementary Table S2), which were designed to amplify the *actin* gene to verify the integrity of the DNA samples. The results revealed none of the 21 passaged transformants were still able to grow in the presence of oxathiapiprolin, and that all of them had lost the *PcMuORP1* transgene (Figure 4). Similar results were obtained for the 32 single zoospore progeny: none of them still harbored the *PcMuORP1* transgene (Supplementary Figure S3). However, it should be noted that the experiment evaluating the loss of the selection marker during asexual reproduction was performed in the absence of selective pressure, so it is uncertain whether the process of asexual reproduction alone is sufficient to result in the loss of the marker. Taken together these results indicate that the selection marker *PcMuORP1* is easily lost from transformants in the absence of selective pressure, and that it would therefore have little long term effect on the fitness of *P. sojae* transformants.

Further experiments were conducted to investigate the stability of the *PcMuORP1* transgene in *P. capsici*. In this case PCR analysis of the three complemented transformants described above and their parental isolate revealed that although the *P. capsici* transformants lost the *PcMuORP1* transgene in the same way as the *P. sojae* transformants, the complemented *PcDHCR7* gene was retained in the asexual progeny (Supplementary Figure S4). These results indicate that the loss of selection markers in the absence of selection pressure might happen in several different *Phytophthora* species, but it would have no influence on target gene editing.

## Discussion

The current study was initiated to evaluate the potential of oxathiapiprolin, which targets the ORP1 protein, as a means of selection during *Phytophthora* transformation. Although proteins belonging to the ORP family have been reported in many organisms including *Homo sapiens* and *Saccharomyces cerevisiae* (Olkkonen, 2013; Charman et al., 2017; Zhao and Ridgway, 2017), their functions remain unknown in oomycetes. However, the ultra-high activity of the fungicide oxathiapiprolin against oomycetes indicates that the target protein ORP1 must be vital to the survival of these species. The results of the study demonstrated that *PcMuORP1* was a very reliable selection marker for the transformation of *P. capsici* and two other *Phytophthora* plant pathogens: *P. sojae* and *P. litchii*, which belong to different clades of *Phytophthora* (Kroon et al., 2012; Ye et al., 2016). It is likely that oxathiapiprolin, which has a relatively wide inhibition spectrum, could be employed as a novel and versatile selective agent for the transformation of a wide range of oomycete species beyond *Phytophthora*.

The current study also demonstrated that *PcMuORP1* was compatible with the CRISPR/Cas9 system, with the three novel plasmids pYF2.3G-PcMuORP1-N, pYF-Cas9-EI, and pB-PcDHCR7 being used to successfully reintroduce the *PcDHCR7* gene into the KD1-1 isolate in which it had been replaced by *NPT II*. This result indicates that under the control of appropriate promoters, *PcMuORP1* may be an extremely versatile selection marker that could be used for a wide range of genetic manipulations including gene silencing, gene overexpression, and the subcellular localization of proteins. Furthermore, previous research has shown that during co-transformation only 4–13% of *P. sojae* transformants screened by G418 contained the non-selectable plasmid (Dou et al., 2008), indicating that the addition of *PcMuORP1* as an alternative selection marker could greatly improve the success rate of co-transformation experiments.

The three-step screening process was found to be extremely stringent and had a 100% success rate, with all of the resulting oxathiapiprolin-resistant transformants being found to contain the *PcMuORP1* transgene. Furthermore, the screening was effective even at low oxathiapiprolin concentrations, which could reduce the cost of transformation. In addition, *PcMuORP1* was found to greatly improve the yield of transformants compared to the conventional selection marker *NPT II*. This effect was especially pronounced in *P. capsici* where a eightfold increase in the number of transformants obtained was observed. Indeed, only a few colonies emerged from the second screening with G418 in spite of being under a lower selective pressure, and of those that did, most failed to grow during the third screening (Figure 3A). These results indicate that *NPT II* is not a very efficient selection marker in *P. capsici*, similar to what was observed when *HPT* was used as a selection marker in *P. sojae*, where some *P. sojae* transformants failed to grow in the third round of the hygromycin B screening (Judelson et al., 1993). Furthermore, the MIC data confirmed that *P. capsici* was insensitive to hygromycin B (Table 1). Taken together, these results confirm that *PcMuORP1* is a competent selection marker for *P. capsici* transformation and that it could therefore greatly improve the progress of functional gene studies in this important pathogen. In addition, the *PcMuORP1* selection marker almost doubled the screening efficiency in *P. sojae* and *P. litchii*. A previous study evaluating the screening efficiency of the selection marker *NPT II* in *P. sojae* found that 30–100 transformants could be produced during the second screening, and that 80% of these survived the third screening (Dou et al., 2008). These Figures are in broad agreement with those obtained in the current study. The higher performance of *PcMuORP1* would therefore not only reduce labor and time, but also further reduce the cost of transformation, and potentially increase the yield of genome editing experiments.

Although selection markers play a critical role in the transformation process, they can sometimes impair the fitness of transformants, and in some cases pose a danger to the environment or human health (Scutt et al., 2002). For example, sometimes high expression levels of selection markers can lead to an excess of redundant proteins in the cell, which can increase the metabolic burden of the transformants (Khan and Maliga, 1999). These risks are particularly prevalent during plant transformation, and several strategies have been developed to eliminate the permanent expression of selection markers in transgenic lines, including the application of site specific recombination systems, transcription activator-like effector nucleases (TALENs), and zinc finger nucleases (ZFNs) (Yau and Stewart, 2013). The current study found that the *PcMuORP1* gene was easily lost in the absence of selective pressure and in the progeny resulting from asexual reproduction. Similar results have been reported in a previous study, which noted the easy loss of drug (G418 and hygromycin B) resistance in asexual progeny of transformants of *P. parasitica* (Gaulin et al., 2007). The reason why the selection marker was so easily lost from the transformants assessed in the current study is uncertain, but possibly it resulted from there being no integration of the plasmid into the genome or that the integration was unstable. However, given that the only purpose of the selection marker is to screen transformants this is not of particular concern. Also, the results in the study demonstrated that the loss of the selection marker would not impair the target gene editing. In fact, this loss of the selection marker could be advantageous as it allows the reuse of the same marker in subsequent transformation projects facilitating complementation or multiplex editing.

A large number of oomycete pathogens have a great economic and ecological impact as a result of the effects they exert on their hosts. However, many aspects of oomycete biology, including plant pathogen interactions, are poorly understood, which makes it difficult to manipulate their behaviors in integrated pest management programs. Although, the genome sequences of more than 20 oomycetes have already been published, with many others in the process of being sequenced (Tyler et al., 2006; Tian et al., 2011; Fang et al., 2017), genome editing is sometimes impeded due to the lack of available selection markers. A broad range of selection markers, which can be categorized according to their modes of action (Bashir et al., 2016), are available for the transformation of different organisms, including antibiotic resistance genes, herbicides resistance genes and auxotroph saving genes (Miki and McHugh, 2004; Kanda et al., 2014; Hu et al., 2016). However, the use of fungicide resistance genes is rarely employed, even though the resistance mechanisms of more and more fungicides have been characterized in detail, including carbendazim, tebuconazole, boscalid, phenamacril, zoxamide, mandipropamid, and oxathiapiprolin (Fujimura et al., 1994; Blum et al., 2010; Cai et al., 2015, 2016; Wang et al., 2015; Zheng et al., 2015; Miao et al., 2016a, 2018; Lichtemberg et al., 2017). The development of these fungicides and their related resistance genes as selection markers could greatly expand the options available for the transformation of oomycetes and true fungi.

## Conclusion

We have created an alternative selection marker for *Phytophthora* transformation by utilizing a fungicide resistance gene. The novel marker was compatible with CRISPR/Cas9 system and could be used in a broad range of *Phytophthora* species. Furthermore, the marker resulted in 100% positive screening rate and much higher screening efficiency compared with *NPT II*. Stability test of the marker showed that it could be lost easily from the transformants under no selective pressure, which indicates that the *PcMuORP1* gene would have little long term influence on the fitness of transformants and could be reused as the selection marker in subsequent projects.

## Data Availability Statement

All datasets generated for this study are included in the manuscript/Supplementary Files.

## Author Contributions

XL and WW conceived and designed the experiments. WW, ZX and JM performed the experiments. MC, CZ, TL, and BZ contributed reagents, materials, and analysis tools. BT gave important suggestions during the process of the work. XL supervised the work. WW wrote the main manuscript. XL and BT revised the manuscript. All authors have read and approved the final manuscript.

## Conflict of Interest

The authors declare that the research was conducted in the absence of any commercial or financial relationships that could be construed as a potential conflict of interest.

## References

[B1] Alonso J. M. Stepanova A. N. Leisse T. J. Kim C. J. Chen H. Shinn P. (2003). Genome-wide insertional mutagenesis of *Arabidopsis thaliana*. *Science* 301 653–657. 10.1126/science.1086391 12893945

[B2] Bailey A. M. Mena G. L. Herrera-Estrella L. (1991). Genetic transformation of the plant pathogens *Phytophthora capsici* and *Phytophthora parasitica*. *Nucleic Acids Res.* 19 4273–4278. 10.1093/nar/19.15.4273 1651483 PMC328573

[B3] Baldauf S. L. Roger A. J. WenkSiefert I. Doolittle W. F. (2000). A kingdom-level phylogeny of eukaryotes based on combined protein data. *Science* 290 972–977. 10.1126/science.290.5493.972 11062127

[B4] Bargmann C. I. (2001). High-throughput reverse genetics: RNAi screens in *Caenorhabditis elegans*. *Genome Biol.* 2:reviews1005. 11182891 10.1186/gb-2001-2-2-reviews1005 PMC138903

[B5] Bashir K. M. I. Kim M. S. Stahl U. Cho M. J. (2016). Microalgae engineering toolbox: selection and screenable markers. *Biotechnol. Bioproc. Eng.* 21 224–235. 10.1007/s12257-015-0386-4

[B6] Blum M. Waldner M. Gisi U. (2010). A single point mutation in the novel *PvCesA3* gene confers resistance to the carboxylic acid amide fungicide mandipropamid in *Plasmopara viticola*. *Fungal Genet. Biol.* 47 499–510. 10.1016/j.fgb.2010.02.009 20226261

[B7] Cai M. Lin D. Chen L. Bi Y. Xiao L. Liu X. (2015). M233I mutation in the β-tubulin of *Botrytis cinerea* confers resistance to zoxamide. *Sci. Rep.* 5:16881. 10.1038/srep16881 26596626 PMC4657022

[B8] Cai M. Miao J. Song X. Lin D. Bi Y. (2016). C239S mutation in the β-tubulin of *Phytophthora sojae* confers resistance to zoxamide. *Front. Microbiol.* 7:762. 10.3389/fmicb.2016.00762 27242773 PMC4873504

[B9] Charman M. Goto A. Ridgway N. D. (2017). Oxysterol binding protein recruitment and activity at the ER-Golgi interface are independent of Sac1. *Traffic* 18 519–529. 10.1111/tra.12491 28471037

[B10] Cvitanich C. Judelson H. S. (2003). Stable transformation of the oomycete, *Phytophthora infestans*, using microprojectile bombardment. *Curr. Genet.* 42 228–235. 12589474 10.1007/s00294-002-0354-3

[B11] Derevnina L. Petre B. Kellner R. Dagdas Y. F. Sarowar M. N. Giannakopoulou A. (2016). Emerging oomycete threats to plants and animals. *Philos. Trans. R. Soc. Lond. B Biol. Sci.* 371:20150459. 10.1098/rstb.2015.0459 28080985 PMC5095538

[B12] Desmond E. Gribaldo S. (2009). Phylogenomics of sterol synthesis: insights into the origin, evolution, and diversity of a key eukaryotic feature. *Genome Biol. Evol.* 1 364–381. 10.1093/gbe/evp036 20333205 PMC2817430

[B13] Dou D. Kale S. D. Liu T. Tang Q. Wang X. Arredondo F. D. (2010). Different domains of *Phytophthora sojae* effector Avr4/6 are recognized by soybean resistance genes Rps4 and Rps6. *Mol. Plant Microbe Interact.* 23 425–435. 10.1094/MPMI-23-4-0425 20192830

[B14] Dou D. Kale S. D. Wang X. Chen Y. Wang Q. Wang X. (2008). Conserved C-terminal motifs required for avirulence and suppression of cell death by *Phytophthora sojae* effector Avr1b. *Plant Cell* 20 1118–1133. 10.1105/tpc.107.057067 18390593 PMC2390733

[B15] Fang Y. Cui L. Gu B. Arredondo F. Tyler B. M. (2017). Efficient genome editing in the oomycete *Phytophthora sojae* using CRISPR/Cas9. *Curr. Protoc. Microbiol.* 44:21A.1.1-21A.1.26. 10.1002/cpmc.25 28166383

[B16] Fang Y. Tyler B. M. (2016). Efficient disruption and replacement of an effector gene in the oomycete *Phytophthora sojae* using CRISPR/Cas9. *Mol. Plant Pathol.* 17 127–139. 10.1111/mpp.12318 26507366 PMC6638440

[B17] Fry W. E. Birch P. R. J. Judelson H. S. Griinwald N. J. Danies G. Everts K. L. (2015). Five reasons to consider *Phytophthora infestans* a reemerging pathogen. *Phytopathology* 105 966–981. 10.1094/PHYTO-01-15-0005-FI 25760519

[B18] Fujimura M. Kamakura T. Inoue H. Yamaguchi I. (1994). Amino-acid alterations in the β-tubulin gene of *Neurospora crassa* that confer resistance to carbendazim and diethofencarb. *Curr. Genet.* 25 418–440. 8082187 10.1007/BF00351780

[B19] Gaulin E. Haget N. Khatib M. Herbert C. Rickauer M. Bottin A. (2007). Transgenic sequences are frequently lost in *Phytophthora parasitica* transformants without reversion of the transgene-induced silenced state. *Can. J. Microbiol.* 53 152–157. 10.1139/w06-090 17496962

[B20] Gumtow R. Wu D. Uchida J. Tian M. (2018). A *Phytophthora palmivora* extracellular cystatin-like protease inhibitor targets papain to contribute to virulence on papaya. *Mol. Plant Microbe Interact.* 31 363–373. 10.1094/MPMI-06-17-0131-FI 29068239

[B21] Hannon G. J. (2002). RNA interference. *Nature* 418 244–251. 12110901 10.1038/418244a

[B22] Hu L. Li H. Qin R. Xu R. Li J. Li L. (2016). Plant phosphomannose isomerase as a selection marker for rice transformation. *Sci. Rep.* 6:25921. 10.1038/srep25921 27174847 PMC4865823

[B23] Ji P. Csinos A. S. Hickman L. L. Hargett U. (2014). Efficacy and application methods of oxathiapiprolin for management of black shank on tobacco. *Plant Dis.* 98 1551–1554. 10.1094/PDIS-02-14-0172-RE 30699789

[B24] Jiang L. Ye W. Situ J. Chen Y. Yang X. Kong G. (2017). A Puf RNA-binding protein encoding gene *PlM90* regulates the sexual and asexual life stages of the litchi downy blight pathogen *Peronophythora litchii*. *Fungal Genet. Biol.* 98 39–45. 10.1016/j.fgb.2016.12.002 27939344

[B25] Judelson H. S. (1997). The genetics and biology of *Phytophthora infestans*: modern approaches to a historical challenge. *Fungal Genet. Biol.* 22 65–76. 10.1006/fgbi.1997.1006 9367653

[B26] Judelson H. S. Blanco F. A. (2005). The spores of *Phytophthora*: weapons of the plant destroyer. *Nat. Rev. Microbiol.* 3 47–58. 10.1038/nrmicro1064 15608699

[B27] Judelson H. S. Coffey M. D. Arredondo F. R. Tyler B. M. (1993). Transformation of the oomycete pathogen *Phytophthora megasperma* f. sp. glycinea occurs by DNA integration into single or multiple chromosomes. *Curr. Genet.* 23 211–218. 10.1007/bf00351498 8382110

[B28] Judelson H. S. Michelmore R. W. (1991). Transient expression of genes in the oomycete *Phytophthora infestans* using *Bremia lactucae* regulatory sequences. *Curr. Genet.* 19 453–459. 10.1007/bf00312736

[B29] Kamath R. S. Martinez-Campos M. Zipperlen P. Fraser A. G. Ahringer J. (2001). Effectiveness of specific RNA-mediated interference through ingested double-stranded RNA in *Caenorhabditis elegans*. *Genome Biol.* 2:research0002.1. 11178279 10.1186/gb-2000-2-1-research0002 PMC17598

[B30] Kamoun S. Furzer O. Jones J. D. G. Judelson H. S. Ali G. S. Dalio R. J. D. (2015). The Top 10 oomycete pathogens in molecular plant pathology. *Mol. Plant Pathol.* 16 413–434. 10.1111/mpp.12190 25178392 PMC6638381

[B31] Kanda K. Ishida T. Hirota R. Ono S. Motomura K. Ikeda T. (2014). Application of a phosphite dehydrogenase gene as a novel dominant selection marker for yeasts. *J. Biotechnol.* 18 68–73. 10.1016/j.jbiotec.2014.04.012 24786825

[B32] Kao C. W. Leu L. S. (1980). Sporangium germination of *Peronophythora litchii*, the causal organism of litchi downy blight. *Mycologia* 72 737–748. 10.1080/00275514.1980.12021242

[B33] Khan M. S. Maliga P. (1999). Fluorescent antibiotic resistance marker for tracking plastid transformation in higher plants. *Nat. Biotechnol.* 17 910–915. 10.1038/12907 10471936

[B34] Kroon L. P. Brouwer H. de Cock A. W. Govers F. (2012). The genus *Phytophthora* anno 2012. *Phytopathology* 102 348–364. 10.1094/PHYTO-01-11-0025 22185336

[B35] Lamour K. H. Stam R. Jupe J. Huitema E. (2012). The oomycete broad-host-range pathogen *Phytophthora capsici*. *Mol. Plant Pathol.* 13 329–337. 10.1111/j.1364-3703.2011.00754.x 22013895 PMC6638677

[B36] Latijnhouwers M. Govers F. (2003). A *Phytophthora infestans* G-protein beta subunit is involved in sporangium formation. *Eukaryot. Cell* 2 971–977. 10.1128/ec.2.5.971-977.2003 14555479 PMC219352

[B37] Lichtemberg P. S. F. Luo Y. Morales R. G. Fischer J. M. M. Michailides T. J. May De Mio L. L. (2017). The point mutation G461S in the *MfCYP51* gene is associated with tebuconazole resistance in *Monilinia fructicola* populations in Brazil. *Phytopathology* 107 1507–1514. 10.1094/PHYTO-02-17-0050-R 28697663

[B38] Liu T. Ye W. Ru Y. Yang X. Gu B. Tao K. (2011). Two host cytoplasmic effectors are required for pathogenesis of *Phytophthora sojae* by suppression of host defenses. *Plant Phys.* 155 490–501. 10.1104/pp.110.166470 21071601 PMC3075790

[B39] Lu S. Chen L. Tao K. Sun N. Wu Y. Lu X. (2013). Intracellular and extracellular phosphatidylinositol 3-phosphate produced by *Phytophthora* species are important for infection. *Mol. Plant* 6 1592–1604. 10.1093/mp/sst047 23475996

[B40] Miao J. Cai M. Dong X. Liu L. Lin D. Zhang C. (2016a). Resistance assessment for oxathiapiprolin in *Phytophthora capsici* and the detection of a point mutation (G769W) in PcORP1 that confers resistance. *Front. Microbiol.* 7:615. 10.3389/fmicb.2016.00615 27199944 PMC4850160

[B41] Miao J. Dong X. Lin D. Wang Q. Liu P. Chen F. (2016b). Activity of the novel fungicide oxathiapiprolin against plant-pathogenic oomycetes. *Pest. Manag. Sci.* 72 1572–1577. 10.1002/ps.4189 26577849

[B42] Miao J. Chi Y. Lin D. Tyler B. M. Liu X. (2018). Mutations in ORP1 conferring oxathiapiprolin resistance confirmed by genome editing using CRISPR/Cas9 in *Phytophthora capsici* and *P. sojae*. *Phytopathology* 108 1412–1419. 10.1094/PHYTO-01-18-0010-R 29979095

[B43] Miki B. McHugh S. (2004). Selection marker genes in transgenic plants: applications, alternatives and biosafety. *J. Biotechnol.* 107 193–232. 10.1016/j.jbiotec.2003.10.011 14736458

[B44] Olkkonen V. M. (2013). OSBP-related proteins: liganding by glycerophospholipids opens new insight into their function. *Molecules* 18 13666–13679. 10.3390/molecules181113666 24196413 PMC6270239

[B45] Pang Z. Shao J. Chen L. Lu X. Hu J. Qin Z. (2013). Resistance to the novel fungicide pyrimorph in *Phytophthora capsici*: risk assessment and detection of point mutations in CesA3 that confer resistance. *PLoS One* 8:e56513. 10.1371/journal.pone.0056513 23431382 PMC3576395

[B46] Pasteris R. J. Hanagan M. A. Bisaha J. J. Finkelstein B. L. Hoffman L. E. Gregory V. (2016). Discovery of oxathiapiprolin, a new oomycete fungicide that targets an oxysterol binding protein. *Bioorgan. Med. Chem.* 24 354–361. 10.1016/j.bmc.2015.07.064 26314923

[B47] Ristaino J. B. Madritch M. Trout C. L. Parra G. (1998). PCR amplification of ribosomal DNA for species identification in the plant pathogen genus *Phytophthora*. *Appl. Environ. Microb.* 64 948–954. 9501434 10.1128/aem.64.3.948-954.1998 PMC106350

[B48] Scutt C. P. Zubko E. Meyer P. (2002). Techniques for the removal of marker genes from transgenic plants. *Biochimie* 84 1119–1126. 10.1016/s0300-9084(02)00021-4 12595140

[B49] Tian M. Win J. Savory E. Burkhardt A. Held M. Brandizzi F. (2011). 454 Genome sequencing of *Pseudoperonospora cubensis* reveals effector proteins with a QXLR translocation motif. *Mol. Plant Microbe Interact.* 24 543–553. 10.1094/MPMI-08-10-0185 21261462

[B50] Tyler B. M. (2007). *Phytophthora sojae*: root rot pathogen of soybean and model oomycete. *Mol. Plant Pathol.* 8 1–8. 10.1111/j.1364-3703.2006.00373.x 20507474

[B51] Tyler B. M. Gijzen M. (2014). “The *Phytophthora sojae* genome sequence: foundation for a revolution,” in *Genomics of Plant-Associated Fungi and Oomycetes: Dicot Pathogens*, eds Dean R. A. Lichens-Park A. Kole C. (New York, NY: Springer Press), 133–157. 10.1007/978-3-662-44056-8_7

[B52] Tyler B. M. Tripathy S. Zhang X. Dehal P. Jiang R. H. Aerts A. (2006). *Phytophthora* genome sequences uncover evolutionary origins and mechanisms of pathogenesis. *Science* 313 1261–1266. 10.1126/science.1128796 16946064

[B53] van West P. Kamoun S. van’t Klooster J. W. Govers F. (1999). Internuclear gene silencing in *Phytophthora infestans*. *Mol. Cell* 3 339–348. 10.1016/s1097-2765(00)80461-x 10198636

[B54] Vijn I. Govers F. (2003). *Agrobacterium tumefaciens* mediated transformation of the oomycete plant pathogen *Phytophthora infestan*. *Mol. Plant Pathol.* 4 459–467. 10.1046/j.1364-3703.2003.00191.x 20569405

[B55] Wang Q. Han C. Ferreira A. O. Yu X. Ye W. Tripathy S. (2011). Transcriptional programming and functional interactions within the *Phytophthora sojae* RXLR effector repertoire. *Plant Cell* 23 2064–2086. 10.1105/tpc.111.086082 21653195 PMC3160037

[B56] Wang Y. Duan Y. Wang J. Zhou M. (2015). A new point mutation in the iron-sulfur subunit of succinate dehydrogenase confers resistance to boscalid in *Sclerotinia sclerotiorum*. *Mol. Plant Pathol.* 16 653–661. 10.1111/mpp.12222 25441450 PMC6638386

[B57] Ward E. W. B. Cahill D. M. Bhattacharyya M. K. (1989). Early cytological differences between compatible and incompatible interactions of soybeans with *Phytophthora megasperma* f. sp. *glycinea*. *Physiol. Mol. Plant. P.* 34 267–283. 10.1016/0885-5765(89)90049-0

[B58] Wu D. Navet N. Liu Y. Uchida J. Tian M. (2016). Establishment of a simple and efficient *Agrobacterium*-mediated transformation system for *Phytophthora palmivora*. *BMC Microbiol.* 16:204. 10.1186/s12866-016-0825-1 27599726 PMC5012004

[B59] Xie K. Minkenberg B. Yang Y. (2015). Boosting CRISPR/Cas9 multiplex editing capability with the endogenous tRNA-processing system. *Proc. Natl. Acad. Sci. U.S.A.* 112 3570–3575. 10.1073/pnas.1420294112 25733849 PMC4371917

[B60] Yau Y. Y. Stewart C. N. (2013). Less is more: strategies to remove marker genes from transgenic plants. *BMC Biotechnol.* 13:36. 10.1186/1472-6750-13-36 23617583 PMC3689633

[B61] Ye W. Wang Y. Shen D. Li D. Pu T. Jiang Z. (2016). Sequencing of the litchi downy blight pathogen reveals it is a *Phytophthora* species with downy mildew-like characteristics. *Mol. Plant Microbe Interact.* 29 573–583. 10.1094/MPMI-03-16-0056-R 27183038

[B62] Yoon H. S. Hackett J. D. Pinto G. Bhattacharya D. (2002). The single, ancient origin of chromist plastids. *Proc. Natl. Acad. Sci. U.S.A.* 99 15507–15512. 10.1073/pnas.242379899 12438651 PMC137747

[B63] Zadoks J. C. (2008). The potato murrain on the European continent and the revolutions of 1848. *Potato Res.* 51 5–45. 10.1007/s11540-008-9091-4

[B64] Zhao K. Ridgway N. D. (2017). Oxysterol-binding protein-related protein 1L regulates cholesterol egress from the endo-lysosomal system. *Cell Rep.* 19 1807–1818. 10.1016/j.celrep.2017.05.028 28564600

[B65] Zheng Z. Hou Y. Cai Y. Zhang Y. Li Y. Zhou M. (2015). Whole-genome sequencing reveals that mutations in myosin-5 confer resistance to the fungicide phenamacril in *Fusarium graminearum*. *Sci. Rep.* 5:8248. 10.1038/srep08248 25648042 PMC5389027

