# Supplementary material for: PcMuORP1, an Oxathiapiprolin-Resistance Gene, Functions as a Novel Selection Marker for Phytophthora Transformation and CRISPR/Cas9 Mediated Genome Editing
Source: Front Microbiol. 2019 Oct 22;10:2402. doi: 10.3389/fmicb.2019.02402 (PMC6821980; doi:10.3389/fmicb.2019.02402)
Supplement: Supplementary file 1 [file Data_Sheet_1.doc]

Supplementary Material

**Supplementary Methods S1. Plasmid construction and preparation**

All the restriction enzymes used in the current study, including *Nhe*I, *Bsa*I, *Cla*I, *Apa*I, *EcoR*I, *EcoR*V, *Pac*I and *Not*I were obtained from NEB (Beijing, China), and the restriction digestions were performed according to the protocol of the manufacturer. The plasmids discussed in the study were constructed using In-Fusion® HD cloning technology (Clontech, Beijing, China) or T4 ligase (TransGen, Beijing, China), while bacterial transformations were performed using Trans1-T1 competent cells (TransGen, Beijing, China), again following the protocols of the manufacturers. The primers for plasmid construction were designed using an online tool (http://www.clontech.com/US/Products/Cloning_and_Competent_Cells/Cloning_Resources/Online_In-Fusion_Tools) with a few manual modifications. The PCR implemented for plasmid construction was carried out using the high-fidelity DNA polymerase FastPfu system (TransGen, Beijing, China). All the plasmids and primers used in the study have been listed in **Supplementary Tables S1** and **S2**, respectively.

For sgRNA expressing plasmid construction, the sgRNA targeting the *PcDHCR7* (or *NPT II*, or *PcERG3*) gene was designed by an online tool (<http://grna.ctegd.uga.edu/>) (Peng and Tarleton, 2015) and cloned to vector pYF2.3G-ribo-sgRNA (Fang and Tyler, 2016) to produce the plasmid pYF2.3G-D (or pYF2.3G-N for *NPT II*, or pYF2.3G-E for *PcERG3*).

The homologous replacement template plasmid pB-D-NPT for replacement of *PcDHCR7* was constructed by simultaneously introduction of three sequences into the *EcoR*V linearized pBluescript II KS+ vector using In-Fusion® cloning strategy, which involved reassembling multiple PCR fragments (Raman and Martin, 2014). The first sequence was 1020 bp in length including upstream 990 bp of the *PcDHCR7* gene; the second sequence was 810 bp including 795 bp of the donor DNA *NPT II*; and the third sequence was 1005 bp including downstream 990 bp of the *PcDHCR7* gene.

For introducing the G2305T mutation, cDNA from wild-type *P. capsici* isolate BYA5 was used as template for RT-PCR to eliminate an intron near the 5’ end of the *PcORP1* gene. The G2305T point mutation was introduced into the *PcORP1* CDS using a modified In-Fusion® cloning strategy. The *PcMuORP1* gene was amplified as two separate fragments using the 2305^th^ nucleotide as a boundary. Two pairs of primers containing the desired site mutation were designed to amplify the two fragments. The first fragment, which was 2334 bp in length, was amplified using the MuORP1-1-F/MuORP1-1-R primer set, while the second, which was 585 bp, was amplified using MuORP1-2-F and MuORP1-2-R. The resulting PCR products contained the desired mutation and overlapping homologous sequences that facilitated the simultaneous introduction of both sequences into the *EcoR*V linearized pBluescript II KS+ vector using the In-Fusion® HD Cloning Kit. The resulting recombinant plasmid pB-PcMuORP1 containing the putative *PcMuORP1* gene was sequenced to confirm that it contained the G2305T mutation.

For introducing the *PcMuORP1* gene into the plasmid pYF2.3G-N or pYF2.3G-E, the current study replaced the *eGFP* gene in pYF2.3G-N or pYF2.3G-E with *PcMuORP1.* The first step involved the amplification of the *PcMuORP1* gene from the pB-PcMuORP1 vector using the MuORP1-F/MuORP1-R primer set, which contained overlapping sequences corresponding to the *Cla*I/*Apa*I linearized plasmid pYF2.3G-N or pYF2.3G-E that facilitated the subsequent cloning using the In-Fusion® HD Cloning Kit. The resulting plasmid, which could express the *PcMuORP1* gene strongly via the Ham34 promoter, was named pYF2.3G-PcMuORP1-N or pYF2.3G-PcMuORP1-E.

The Cas9 expression vector pYF2-PsNLS-hSpCas9 used in the original replacement study (Fang and Tyler, 2016), which also contained the *NPT II* gene was also modified to prevent any interference with the sgRNA-N targeting in complemention project. In this case the entire *NPT II* expression cassette was removed by *EcoR*I digestion (Each side of the *NPT II* expression cassette has a *EcoR*I restriction enzyme cutting site) and T4 ligation to create the novel plasmid pYF-Cas9-EI.

The homologous replacement template for the *PcDHCR7* complementation was created by cloning the entire *PcDHCR7* gene together with its 990 bp upstream and downstream sequences, which was amplified by the D-up-F/D-down-R primer set, into pBluescript II KS+ as a *EcoR*V restriction fragment to generate the vector pB-PcDHCR7.

The homologous replacement template plasmid pB-E-NPT for replacement of *PcERG3* was constructed by simultaneously introduction of three sequences into the *EcoR*V linearized pBluescript II KS+ vector using the In-Fusion® HD Cloning Kit. The first sequence was 978 bp in length including upstream 948 bp of the *PcERG3* gene, which was amplified by the E-up-F/E-up-R primer set; the second sequence was 810 bp including 795 bp of the donor DNA *NPT II*, which was amplified by the E-NPT-F/E-NPT-R primer set; and the third sequence was 967 bp including downstream 952 bp of the *PcERG3* gene, which was amplified by the E-down-F/E-down-R primer set.

To construct a backbone vector containing PcMuORP1, Cas9 and sgRNA expressing cassettes, which could be used for cloning sgRNA fragment directly, the all-in-one vector pYF515 (Fang et al., 2017) was modified by replacing the *NPT II* gene with the *PcMuORP1*. To begin with, the restriction enzyme site analysis was conducted using SnapGene® Viewer 3.0.3, and the result showed that the *PcMuORP1* sequence contained the *Nhe*I and *Bsa*I restriction enzyme sites which were used for sgRNA fragment cloning (**Supplementary Figure S2**). Therefore, two nonsense mutations were introduced into the *PcMuORP1* sequence to eliminate the *Nhe*I and *Bsa*I restriction enzyme sites. Briefly, the original all-in-one vector pYF515 was linearized by *Pac*I/*Not*I digestion, and the nonsense mutated *PcMuORP1* was amplified from the plasmid pYF2.3G-PcMuORP1-N, which was divided into three separate fragments with NheI/BsaI restriction enzyme sites as boundaries. Three pairs of primers containing the desired site mutations were designed. The first fragment, which was 1580 bp in length, was amplified using the NonORP1-F1/NonORP1-R1 primer set; the second, which was 868 bp, was amplified using NonORP1-F2/NonORP1-R2 primer set; and the third, which was 486 bp, was amplified using NonORP1-F3/NonORP1-R3 primer set. All three fragments contained desired mutations and overlapping sequences with the neighbour fragments. After DNA electrophoresis analysis and gel extraction, the fragments were simultaneously cloned into the sgRNA expression vector by In-Fusion® HD Cloning Kit, resulting in a new vector pYF515-PcMuORP1 which contains the nonsense mutated *PcMuORP1*.

For construction of the plasmid pYF2.3G-NPT-N which was equivalent to pYF2.3G-PcMuORP1-N but containing the selectable marker *NPT II*, the pYF2.3G-N vector was first linearized by *Cla*I/*Apa*I (NEB) digestion before the *NPT II* gene was amplified using primer set sg-NPT-F/sg-NPT-R and cloned into the sgRNA expression vector using the In-Fusion® HD Cloning Kit.

All of the *Escherichia coli* strains carrying the plasmid constructs were screened using solid LB medium modified with 100 μg/ml ampicillin, and validated by PCR and sequence analysis (Tsingke, Beijing, China). The plasmids for transformation were then recovered from ampicillin (100 μg/ml)-amended LB liquid cultures of the validated *E. coli* strains incubated at 37°C with shaking (200 rpm) for 14 -18 hours using the EndoFree Maxi Plasmid Kit (Tiangen, Beijing, China).

**References**

Fang, Y., and Tyler, B. M. (2016). Efficient disruption and replacement of an effector gene in the oomycete *Phytophthora sojae* using CRISPR/Cas9. *Mol. Plant Pathol.* 17, 127-139.

Fang, Y., Cui, L., Gu, B., Arredondo, F., and Tyler, B. M. (2017). Efficient genome editing in the oomycete *Phytophthora sojae* using CRISPR/Cas9. *Curr. Protoc. Microbiol.* 44:21A. 1(1-26).

Peng, D., and Tarleton, R. (2015). EuPaGDT: a web tool tailored to design CRISPR guide RNAs for eukaryotic pathogens. *Microb. Genom.* 1:e000033.

Raman, M., and Martin, K. (2014). One solution for cloning and mutagenesis: In-Fusion (R) HD Cloning Plus. *Nat. Methods* 11, III-V.

**Supplementary Methods S2. Replacing *PcDHCR7* and *PcERG3* with *NPT II***

The *PcDHCR7* gene of *P. capsici* (protein ID: 534197 in JGI database), was replaced by the [exogenous](../../../../C:/Users/wang%20weizhen/AppData/Local/Youdao/Dict/Application/8.1.2.0/resultui/html/index.html#/javascript:;) [gene](../../../../C:/Users/wang%20weizhen/AppData/Local/Youdao/Dict/Application/8.1.2.0/resultui/html/index.html#/javascript:;) *NPT II*, using the method described by Fang et al. (2017). Three plasmids, pYF2.3G-D, which contained the sgRNA; pYF2-PsNLS-hSpCas9, which expressed the Cas9 protein and NPT II protein; pB-D-NPT, which served as the homologous replacement template, were simultaneously transformed into protoplasts of BYA5. The transformants were obtained by G418 screening and confirmed by PCR analysis, and gene sequencing of the knock-out transformants including KD1-1 showed that the genome editing was accurate.

The *PcERG3* gene of *P. capsici* (protein ID: 37754 in JGI database), was replaced by the [exogenous](../../../../C:/Users/wang%20weizhen/AppData/Local/Youdao/Dict/Application/8.1.2.0/resultui/html/index.html#/javascript:;) [gene](../../../../C:/Users/wang%20weizhen/AppData/Local/Youdao/Dict/Application/8.1.2.0/resultui/html/index.html#/javascript:;) *NPT II*, using *PcMuORP1* as the selection marker. Three plasmids, pYF2.3G-PcMuORP1-E, which contained the sgRNA and the selection marker *PcMuORP1*; pYF2-Cas9-EI, which expressed the Cas9 protein; pB-E-NPT, which served as the homologous replacement template, were simultaneously transformed into protoplasts of BYA5. The transformants were obtained by oxathiapiprolin screening and 21 randomly selected transformants were used for PCR analysis. The result showed that replacement had occured in 10 of the 21 transformants, among which 3 replacement transformants were homozygous (**Supplementary Figure S1**). Also gene sequencing of the knock-out transformants showed that the genome editing was accurate.

**References**

Fang, Y., Cui, L., Gu, B., Arredondo, F., and Tyler, B. M. (2017). Efficient genome editing in the oomycete *Phytophthora sojae* using CRISPR/Cas9. *Curr. Protoc. Microbiol.* 44:21A. 1(1-26).

**Supplementary Table S1. Plasmids used in the current study**

| **Plasmid name** | **Vector** | **Purpose** |
| --- | --- | --- |
| pB-PcMuORP1 | pBluescript II KS+ | Cloning the *PcMuORP1* gene to introduce the G2305T point mutation |
| pYF2.3G-D | pYF2.3G-ribo-sgRNA | Expression of sgRNA for targeting *PcDHCR7* |
| pYF2.3G-N | pYF2.3G-ribo-sgRNA | Expression of sgRNA for targeting *NPT II* |
| pYF2.3G-PcMuORP1-N | pYF2.3G-N | Expression of sgRNA for targeting *NPT II*, and expression of the *PcMuORP1* gene |
| pYF2.3G-E | pYF2.3G-ribo-sgRNA | Expression of sgRNA for targeting *PcERG3* |
| pYF2.3G-PcMuORP1-E | pYF2.3G-E | Expression of sgRNA for targeting *PcERG3*, and expression of the *PcMuORP1* gene |
| pYF2.3G-NPT-N | pYF2.3G-N | Expression of the *NPT II* gene |
| pYF-Cas9-EI | pYF2-PsNLS-hSpCas9 | Expression of the Cas9 protein during gene complementation |
| pB-D-NPT | pBluescript II KS+ | Homology-directed repair (HDR)-mediated replacement of the *PcHDCR7* gene with *NPT II* |
| pB-PcDHCR7 | pBluescript II KS+ | Homology-directed repair (HDR)-mediated replacement of the *NPT II* gene with *PcHDCR7* |
| pB-E-NPT | pBluescript II KS+ | Homology-directed repair (HDR)-mediated replacement of the *PcERG3* gene with *NPT II* |
| pYF515-PcMuORP1 | pYF515 | All-in-one backbone plasmid containing PcMuORP1, Cas9 and sgRNA expressing cassettes |

**Supplementary Table S2. Primers used in the current study**

| **Primer name** | **Primer sequence 5'-3'** | **Purpose** | **Annealing temperature (ºC)** |
| --- | --- | --- | --- |
| MuORP1-1-F | ATCGATAAGCTTGATATGCAGGCGCTTCAGGAC | Amplification of the first fragment of the PcMuORP1 gene | 58 |
| MuORP1-1-R | AACACGCACAGGCCACTTGTTGGTGTTGAGCATAG | Amplification of the first fragment of the PcMuORP1 gene and to introduce the G2305T point mutation | 58 |
| MuORP1-2-F | TGGCCTGTGCGTGTTACGTTCCCTGACGCGGAT | Amplification of the second fragment of the PcMuORP1 and to introduce the G2305T point mutation | 58 |
| MuORP1-2-R | CTGCAGGAATTCGATCTAATGCCCAGCCGAACT | Amplification of the second fragment of the PcMuORP1 gene | 58 |
| M13-F | TGTAAAACGACGGCCAGT | Confirmation that the relevant sequences had been successfully introduced into the pBluescript II KS+ vector, and the pYF2.3G-ribo-sgRNA vector | 55 |
| M13-R | CAGGAAACAGCTATGACC | Confirmation that the relevant sequences had been successfully introduced into the pBluescript II KS+ vector | 55 |
| sgRNA-D-F | CTAGCAAAGACCTGATGAGTCCGTGAGGACGAAACGAGTAAGCTCGTC**GTCTTTGAACTTTATCCGGG** | Cloning the sgRNA-D targeting the PcDHCR7 gene into the sgRNA expression vector pYF2.3G-ribo-sgRNA | - |
| sgRNA-D-R | AAACCCCGGATAAAGTTCAAAGACGACGAGCTTACTCGTTTCGTCCTCACGGACTCATCAGGTCTTTG | Cloning the sgRNA-D targeting the PcDHCR7 gene into the sgRNA expression vector pYF2.3G-ribo-sgRNA | - |
| RPL41-Pseq-F | CAAGCCTCACTTTCTGCTGACTG | Confirmation of sgRNA insertion into the sgRNA expression vector pYF2.3G-ribo-sgRNA, and sgRNA sequencing | 55 |
| D-up-F | ATCGATAAGCTTGATTTTGCAGTGCCTTCAGCAC | Amplification of the 990 bp upstream sequence of PcDHCR7 gene, and cloning the amplified fragment into the pBluescript II KS+ vector | 58 |
| D-up-R | ATCTTGTTCAATCATATATATCGGAGACTTTGAAGC | Amplification of the 990 bp upstream sequence of PcDHCR7 gene, and cloning the amplified fragment into the pBluescript II KS+ vector | 58 |
| D-NPT-F | ATGATTGAACAAGATGGATTG | Amplification of the *NPT II* gene as donor DNA for *PcDHCR7* replacement and cloning the amplified fragment into the pBluescript II KS+ vector | 58 |
| D-NPT-R | GTTGAAGTTGCCTTTTCAGAAGAACTCGTCAAGAA | Amplification of the *NPT II* gene as donor DNA for *PcDHCR7* replacement and cloning the amplified fragment into the pBluescript II KS+ vector | 58 |
| D-down-F | AAAGGCAACTTCAACTTGAC | Amplification of the 990 bp downstream sequence of PcDHCR7 gene, and cloning the amplified fragment into the pBluescript II KS+ vector | 58 |
| D-down-R | CTGCAGGAATTCGATGACACTGGACTGGGCTTA | Amplification of the 990 bp downstream sequence of PcDHCR7 gene, and cloning the amplified fragment into the pBluescript II KS+ vector | 58 |
| sgRNA-N-F | CTAGCTGATGGCTGATGAGTCCGTGAGGACGAAACGAGTAAGCTCGTCCCATCATGGCTGATGCAATG | Cloning the sgRNA-N targeting the NPT II gene into the sgRNA expression vector pYF2.3G-ribo-sgRNA | - |
| sgRNA-N-R | AAACCATTGCATCAGCCATGATGGGACGAGCTTACTCGTTTCGTCCTCACGGACTCATCAGCCATCAG | Cloning the sgRNA-N targeting the NPT II gene into the sgRNA expression vector pYF2.3G-ribo-sgRNA | - |
| MuORP1-F | ATATCAAGCTTATCGATATGCAGGCGCTTCAGGAC | Cloning the oxathiapiprolin resistance gene PcMuORP1 into the sgRNA expression vector pYF2.3G-N or pYF2.3G-E | 58 |
| MuORP1-R | TAGGCTCCGGAACCGGGCCCCTAATGCCCAGCCGAACT | Cloning the oxathiapiprolin resistance gene PcMuORP1 into the sgRNA expression vector pYF2.3G-N or pYF2.3G-E | 58 |
| sgRNA-E-F | CTAGCATATCCCTGATGAGTCCGTGAGGACGAAACGAGTAAGCTCGTC**GGATATCTACTCTACTTCAT** | Cloning the sgRNA-E targeting the PcERG3 gene into the sgRNA expression vector pYF2.3G-ribo-sgRNA | - |
| sgRNA-E-R | AAACATGAAGTAGAGTAGATATCCGACGAGCTTACTCGTTTCGTCCTCACGGACTCATCAGGGATATG | Cloning the sgRNA-E targeting the PcERG3 gene into the sgRNA expression vector pYF2.3G-ribo-sgRNA | - |
| E-up-F | ATCGATAAGCTTGATGTGATATCGTCTCGAATTTC | Amplification of the 948 bp upstream sequence of PcERG3 gene, and cloning the amplified fragment into the pBluescript II KS+ vector | 58 |
| E-up-R | ATCTTGTTCAATCATCTAGCGCATAGTAGTCGG | Amplification of the 948 bp upstream sequence of PcERG3 gene, and cloning the amplified fragment into the pBluescript II KS+ vector | 58 |
| E-NPT-F | ATGATTGAACAAGATGGATTGCACGCAGGTTCTCC | Amplification of the *NPT II* gene as donor DNA for *PcERG3* replacement and cloning the amplified fragment into the pBluescript II KS+ vector | 58 |
| E-NPT-R | TTTCTTGATCATGGCTCAGAAGAACTCGTCAAGAA | Amplification of the *NPT II* gene as donor DNA for *PcERG3* replacement and cloning the amplified fragment into the pBluescript II KS+ vector | 58 |
| E-down-F | GCCATGATCAAGAAATGAATGAACATGTATGGC | Amplification of the 952 bp downstream sequence of PcERG3 gene, and cloning the amplified fragment into the pBluescript II KS+ vector | 58 |
| E-down-R | CTGCAGGAATTCGATGGATCGAGACCCCTCGTG | Amplification of the 952 bp downstream sequence of PcERG3 gene, and cloning the amplified fragment into the pBluescript II KS+ vector | 58 |
| KERG3-F | GCCAAATGACGGAACAAAG | Confirmation that replacement of the gene PcERG3 had occurred in oxathiapiprolin-resistant transformants | 58 |
| KERG3-R | CAATAGCAGCCAGTCCCTT | Confirmation that replacement of the gene PcERG3 had occurred in oxathiapiprolin-resistant transformants | 58 |
| PcERG3-F | GACCTCATCCTGGAATACG | Amplification of the *PcERG3* gene in *PcERG3* knock-out transformants to determine their heterozygous/homozygous status | 58 |
| PcERG3-R | TTGTTGTGCGAAAGGTAAT | Amplification of the *PcERG3* gene in *PcERG3* knock-out transformants to determine their heterozygous/homozygous status | 58 |
| NonORP1-F1 | ACAAACTAGCTCTTAATGCAGGCGCTTCAGGAC | Amplification of the first fragment of the nonsense mutated PcMuORP1 | 56 |
| NonORP1-R1 | CGCGTGGTAGGCGCAAGCCACCCCTTCATGTCC | Amplification of the first fragment of the nonsense mutated PcMuORP1 and to introduce the first nonsense point mutation | 56 |
| NonORP1-F2 | TGCGCCTACCACGCGCCAGAGTTTCTTGCTCT | Amplification of the second fragment of the nonsense mutated PcMuORP1 and to introduce the first nonsense point mutation | 56 |
| NonORP1-R2 | ATCCACCGTTCGGTCACCCCAAAGCAAACCACC | Amplification of the second fragment of the nonsense mutated PcMuORP1 and to introduce the second nonsense point mutation | 56 |
| NonORP1-F3 | GACCGAACGGTGGATATCATGGGCAACATGGT | Amplification of the third fragment of the nonsense mutated PcMuORP1 and to introduce the second nonsense point mutation | 56 |
| NonORP1-R3 | GGCGCGCCTGCGGCCCTAATGCCCAGCCGAACTGC | Amplification of the third fragment of the nonsense mutated PcMuORP1 | 56 |
| sg-NPT-F | ATATCAAGCTTATCGATGATTGAACAAGATGGATTG | Cloning the G418 resistance gene NPT II into the sgRNA expression vector pYF2.3G-N | 56 |
| sg-NPT-R | TAGGCTCCGGAACCGTCAGAAGAACTCGTCAAGAAGG | Cloning the G418 resistance gene NPT II into the sgRNA expression vector pYF2.3G-N | 56 |
| Resg-F | ACTCGCCCACGATCGGAAGG | Amplification and sequencing of the PcMuORP1 and NPT II genes to confirm they were successfully cloned into the pYF2.3G-N or pYF2.3G-E vector | 60 |
| Resg-R | CACAAAATCTGCAACTTCGC | Amplification and sequencing of the PcMuORP1 and NPT II genes to confirm they were successfully cloned into the pYF2.3G-N or pYF2.3G-E vector | 60 |
| ReD-F | AGCGATCCAGACCGAGCGATTTCAC | Confirmation that complementation of the gene PcDHCR7 had occurred in oxathiapiprolin-resistant transformants and determination of their heterozygous/homozygous status | 58 |
| ReD-R1 | TTACAGGATCTTTGGCAGGATG | Confirmation that complementation of the gene PcDHCR7 had occurred in oxathiapiprolin-resistant transformants | 58 |
| ReD-R2 | CTCAGAGTATCAAGAGCAGG | Determination of the heterozygous/homozygous status of oxathiapiprolin-resistant transformants | 58 |
| Resg-ORP1-F | ACTCGCCCACGATCGGAAGG | Amplification of the oxathiapiprolin resistance gene PcMuORP1 to confirm its presence in oxathiapiprolin-resistant transformants | 60 |
| Resg-ORP1-R | CTAATGCCCAGCCGAACTGC | Amplification of the oxathiapiprolin resistance gene PcMuORP1 to confirm its presence in oxathiapiprolin-resistant transformants | 60 |
| Ps-Actin-F | CCACGAGGGCGAGCACCATG | Amplification of the actin gene in P. sojae | 62 |
| Ps-Actin-R | GTCTTGTCGGGCAGCTCGTA | Amplification of the actin gene in P. sojae | 62 |

Bold nucleotides indicate the sgRNA sequence, while underlined nucleotides indicate the overlapping sequences required to join DNA fragments during In-Fusion® HD cloning.


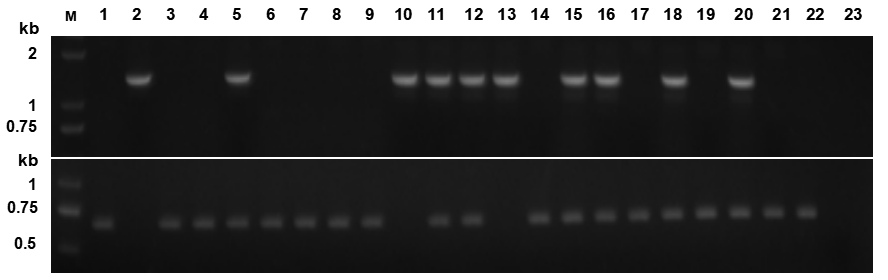


**Supplementary Figure S1. DNA electrophoresis of PCR products amplified from potential *PcERG3* knock-out transformants.** Top: The KERG3-F/KERG3-R primer set confirms replacement has occurred in lanes 2, 5, 10, 11, 12, 13, 15, 16, 18 and 20. Bottom: *PcERG3*-specific primer set (PcERG3-F/PcERG3-R) confirms homozygous replacement in lanes 2, 10 and 13. M: DNA Marker, Lanes 1 to 21: oxathiapiprolin-resistant transformants, Lane 22: Wild-type isolate BYA5, Lane 23: Blank control.


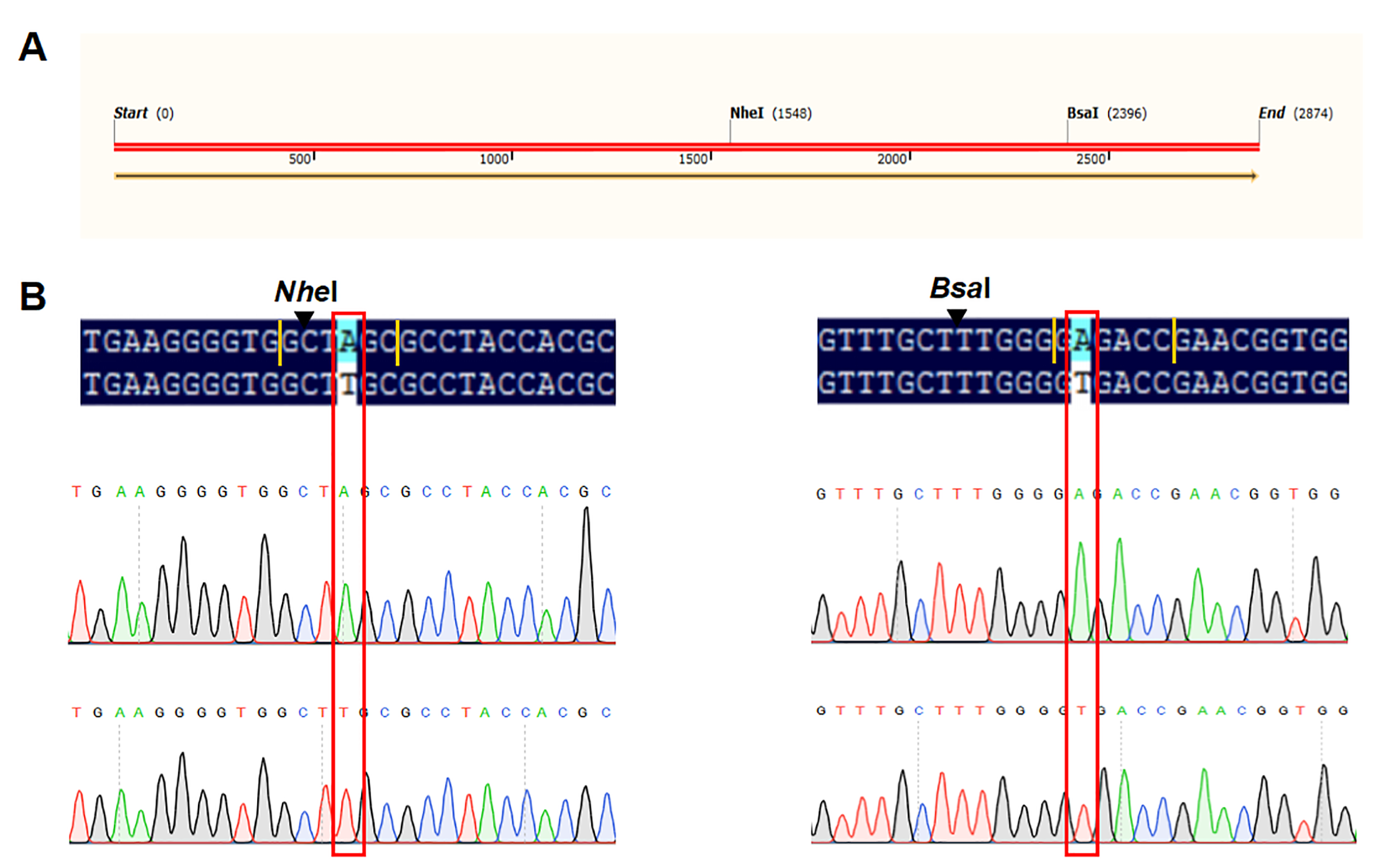


**Supplementary Figure S2.** **Restriction enzyme site analysis of *PcMuORP1* sequence and confirmation of nonsense mutations.** (**A**) Sequence analysis of *PcMuORP1* identified the *Nhe*I and *Bsa*I restriction enzyme sites, which were used for sgRNA fragment cloning. (**B**) Sequence data and multiple sequence alignment confirm that the nonsense point mutations were introduced into the *PcMuORP1* and that the *Nhe*I and *Bsa*I restriction enzyme sites were successfully eliminated. The nonsense mutated nucleotides are shown in different colors, and the sequences recognized by restriction enzymes are shown between yellow bars in the sequence alignment figures. The inverted triangle indicates the cleavage site corresponding to restriction enzyme.


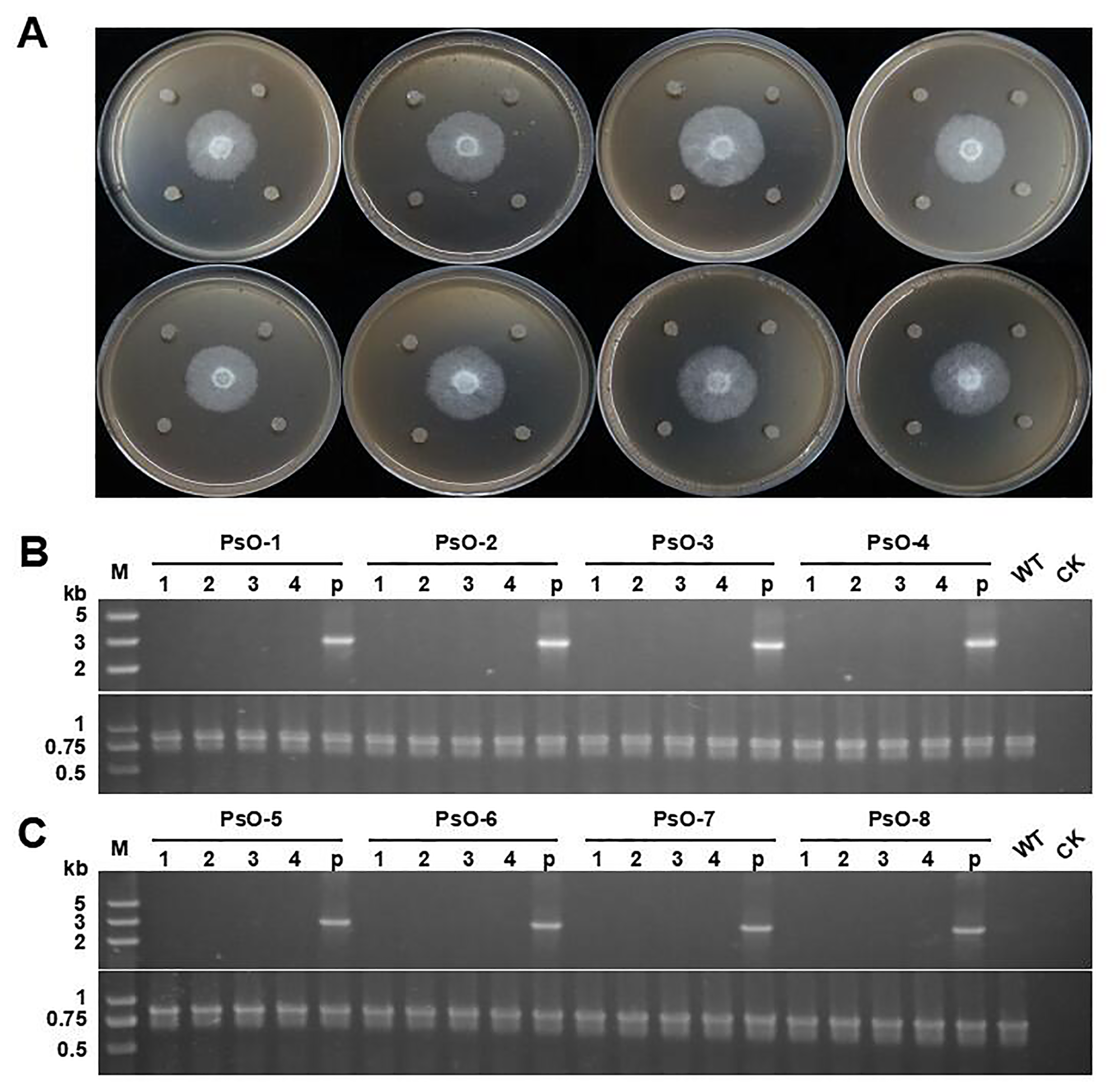


**Supplementary Figure S3.** **Stability of the *PcMuORP1* selection marker in 8 *P. sojae* transformants after single zoospore isolation.** **(A)** Growth of the 32 single zoospore progeny on V8 media amended with 0.01 μg/ml oxathiapiprolin after three days dark-incubation at 25°C. The colony in the center of each plate corresponds to the parental isolate used as a positive control. **(B)** and **(C)** PCR analysis for the detection of the *PcMuORP1* gene. Top: *PcMuORP1*-specific primers. Bottom: *actin*-specific primers. PsO-1 to 8: 8 oxathiapiprolin-resistant transformants with 1, 2, 3 and 4 corresponding to the progeny from 4 different spores, while the lowercase p indicates the parental transformant maintained on oxathiapiprolin-amended V8 medium used as a positive control, WT: Wild-type isolate P6497 used as a negative control, CK: Blank control.


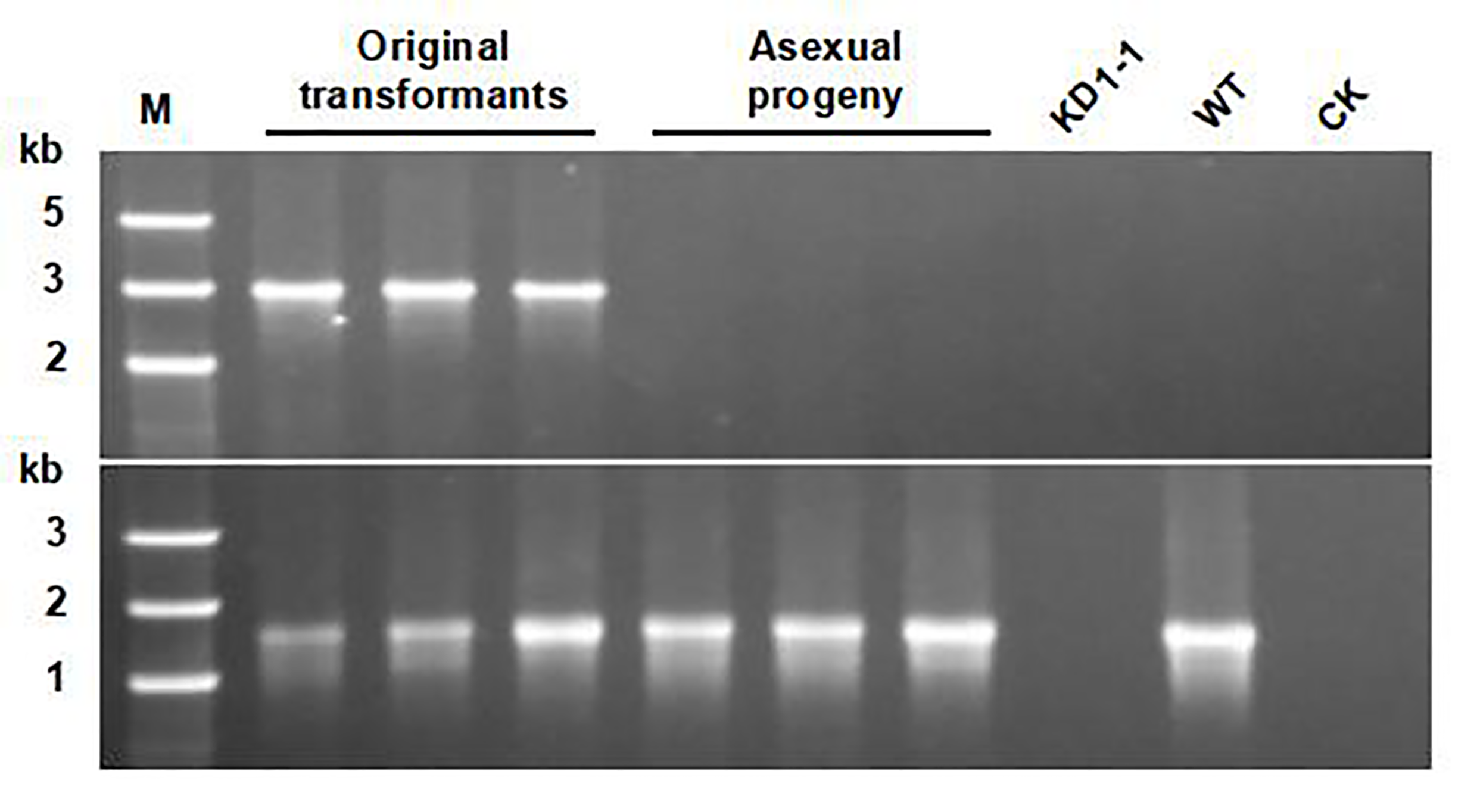


**Supplementary Figure S4.** **PCR analysis to detect the *PcMuORP1* and *PcDHCR7* genes in *P. capsici*.** Top: *PcMuORP1*-specific primers. Bottom: *PcDHCR7*-specific primers. Original transformants: three isolates in which the *PcDHCR7* gene was successfully complemented, Asexual progeny: three isolates obtained by single zoospore purification from three original transformants (one progeny for each). KD1-1: Parental isolate used as a negative control for the detection of *PcDHCR7*, WT: Wild-type isolate BYA5 used as a positive control for the detection of *PcDHCR7*, CK: Blank control.

**>*PcMuORP1* DNA sequence**

1 ATGCAGGCGCTTCAGGACGCGCAACAGCGCTTCAATGACCTCGTGAATAATGACTGGCCGGAACGAGTACCTGTCGAGTC

81 CATGCCGGACTACGACCCGACTTACATGAAGGAAGGCTTCTTGCAGAAGAAAGGACAGAGACTGAAAGGCTGGAAACGCC

161 GCTGGTTTGTCTGTGACGGACGTACACTGTCCTACTACATTTCCAGGAAGGACCGTAAGCCTAATGCTGTCATCCCCTTG

241 GAAGGATGCACCGTGCAGGACGGAGGCCTGAGTGAGACGTGGAACTCGCCTCGTATCTACCTGACGGACCCCGCCACTGG

321 CATCATGTACTGCCTGTCAGCTGAAGAAGGGATTGTAGTCACTCAGTGGCTCGATGTCCTTCGAGTGGCAGTGGCTCGTG

401 TTAATAATGGTACAGCAGCTACTGATTCAAGTGCAGCTCCGTCATCGCAGAGCTCCAGTCACAACAGGACAAGGCAGCAG

481 GCCAGGACGCAGGCTCAGAGACTCCCGTCGTCGTCGGATGATGAAGATTCTCGTGCGCATTTAAAGCGCGCAGCATCCCT

561 GGGACCGTCACAGGCACGTACAACTACGTTGAAGTCGGCTTCGTCGGCTGTGGTCAGTTCCAGTGCAGGCAATTCCACGT

641 CGAATGGAGATGGGAAACGTTCTACAAGGATGACGTCGGCCCCGTCAGCAGTTACCACCTCAAACAGTAATTCACAGCAC

721 ACTCAACACCACCGTGTTCACCGCACGAAGACTCAGCGTCTACCTACGACGATTTCACTGGAGAACGAGCTCTCGCATGG

801 CTTGGATGTGCTGGAGGCGCTGCTGTGTGGCCACAGTGCGACGCGATCTTCGTCTTCACTCCGCAATCACGTCGTGTTTC

881 GGCCTATTGGCGCGGTGAATGGTGTTTTACGCAGCATTGGAACAGATTCGAGTTCTGGGAAGCAATACGCACGTGCTAGT

961 GTGGTGCTCCCGGTGTCTTCAGAAGTAGCGGCGATTCTACTGGCTGACCATGCGCGCAGAGCTGAATGGGACGTGCATTT

1041 TCCTCAATCGGCACATGTTGCTACATTCGATGACGCGACGAATCTCGTGCATTTGTCAAGTGGAAGCTTTGCCCAGATCC

1121 AACAGACCAAGCCAATTGTGGCTCCTCATGTGGCTGCTGCTGCGTGTGCCTTGTGTGCAGCACTCTTCTCTGGTGCTTCA

1201 TCTTGGGAGGCGCTGCTGACTGCGATGATCTACGCTGCAGCCGTTGGTGGTATCGTGAGTAGCATCGACTATAGCACTTT

1281 GACCGCACCTCGCGACCTCGTAGTTCTTCGTCATGTGCGTGAGTCTGCTACGCCTGATTCACAGGATTCAAGCGACGACA

1361 AGTCAGCGGACGAGATGGGCCAGTCAGTGGTGCTTATCCTGGAGAAGTCCGTCGTTAACGAGCTCAAACCTGTTGTCTCC

1441 GGTATTATGCGCGCTCACGTCGGGCTGAGTGGCTGGTTGCTCGAGCCGGTTGATTCAGGTCACGCTACGCTGGCCACCTA

1521 CATTACCGACCTGGACATGAAGGGGTGGCTAGCGCCTACCACGCGCCAGAGTTTCTTGCTCTCCCGTTTGGACTGTGTTT

1601 CAGTTCTAAGCGAGTACGTGAACCAGGCACACTTGTGTGGATCGGAGCTAGGATTTGGTGGTGGTCTTGACGAGGACGGA

1681 GAAGGAGAGTTCGAAACTCGTAGCGTCGGGTACGACGACACTAGCGAAGCAAGCGGTCTAGGAGACGTTGTAGACGGCGA

1761 ATCTTCATCGATCTTCCACCCGAAGACGTACATGCGCGGTATGATGCCGCTTCCGAGCGGTGGACTGAAGCTGATCGACA

1841 AGGAAATCGCTAAGAAACAGGGTGGCGTGGTGAAGGATGTGATCAAATCTGCAGGAGCTAAGATCCTGGAAGGCAAGTCA

1921 GCTGTGAGTCTGTCGCTGCCTGTGCGTATTTTCGAGCCTCGTACGAATCTGGAGCGTGTATGCGACTTGATGCTATACGC

2001 GCCGACGTTTCTAAACGTCGCGTATGCGCAGAATGACGCACTGGAGCGCTTCAAGTACGTAGTGACTTTTGCTGTGGCTG

2081 GACTGCACCACAGTATCGGACAGCTGAAACCATTCAACCCGATCCTGGGCGAAACATACCAGTCCACGCTGAACGACGGC

2161 ACCGACGTTAGCTGTGAACACACGAGCCACCACCCACCTATCAGTAACTTCCAGTTCACGGGTGAAAAGTACTCCATTGC

2241 TGGGTTTGTGCTCTGGCATGCCAGTATGAGCGTCAAGTCCAACGCTATGCTCAACACCAACAAGTGGCCTGTGCGTGTTA

2321 CGTTCCCTGACGCGGAAGGCCTCCCAGGAACAACCATCGAGTACAACCTGCCCTATTTGCAGATCGGTGGTTTGCTTTGG

2401 GGAGACCGAACGGTGGATATCATGGGCAACATGGTGTTTGAAGACAAGAAGAACCGTCTCCAGTGTGAACTGCGTCTCAA

2481 CCCGGATGCGAAGTCGGGTATGGGCGGCATGTTTTCGAGCTCCAAGACCCCCACCGACTCTCTGCGAGGTGTGATCTTGG

2561 ATACTTCCGTGTCTCCTCCACGTGAGATCTGCGATGTGTCCGGTTCGTGGCTGCACGACCTCGTCTTTGGCAACAAGACG

2641 TACTGGAGCATCAACAAGTACCAGAGCGGCTACATGGTGCCGTTCCCTGAGGACAAGATCCTAGCGTCCGACTCTAGACA

2721 CCGTGAAGATTTGCACTATCTGGCTGCAGGCGACTTGGACGAATCGCAAGAGTGGAAGGTGAAGCTGGAGGTGTTGCAGC

2801 GCGCGGACCGCAAGGCACGTCTGGACGGCCGACGTCCCAACCACTGGTCCTTCCGCAGTTCGGCTGGGCATTAG
